# Germline transmission of cryopreserved mouse spermatogonial stem cells maintained on the International Space Station

**DOI:** 10.1016/j.stemcr.2025.102602

**Published:** 2025-08-14

**Authors:** Mito Kanatsu-Shinohara, Takuya Yamamoto, Yusuke Shiromoto, Hiroko Morimoto, Tianjiao Liu, Tohru Yamamori, Tomokazu Yamasaki, Takashi Shinohara

**Affiliations:** 1 Department of Molecular Genetics, Graduate School of Medicine, Kyoto University, Kyoto 606-8501, Japan; 2 AMED, 1-7-1 Otemachi, Chiyodaku, Tokyo 100-0004, Japan; 3 Department of Life Science Frontiers, Center for iPS Cell Research and Application, Kyoto University, Kyoto 606-8507, Japan; 4 Institute for the Advanced Study of Human Biology (WPI-ASHIBi), Kyoto University, Yoshida-Konoe, Sakyo, Kyoto 606-8501, Japan; 5 Medical-risk Avoidance based on iPS Cells Team, RIKEN Center for Advanced Intelligence Project (AIP), Kyoto 606-8507, Japan; 6 Space Utilization Promotion Department, Japan Space Forum, Tokyo 101-0062, Japan; 7 Japan Aerospace Exploration Agency, Tsukuba 305-8505, Japan

**Keywords:** cryopreservation, spaceflight, spermatogonial stem cells, transplantation

## Abstract

Despite growing interest in space exploration, the effects of spaceflight on stem cells remain largely unknown. Damages to germline cells are especially crucial, as spaceflight poses risks to gametogenesis, with abnormalities observed in multiple species. While embryonic stem (ES) cells show genomic instability after space exposure and have not yet produced offspring, this study demonstrates successful offspring production from cryopreserved mouse spermatogonial stem cells (SSCs) stored on the International Space Station for 6 months. Spaceflight did not increase apoptosis or DNA damage in SSCs. After thawing, SSCs proliferated comparably to those cryopreserved on Earth, showing no significant phenotypic or functional differences. Offspring were produced via spermatogonial transplantation followed by natural mating. Since SSCs from many species can be cryopreserved like somatic cells and still produce sperm, they offer a promising resource for germline preservation during space exploration.

## Highlights

• Frozen SSCs were relatively resistant to irradiation
• Frozen SSCs restored spermatogenesis following spaceflight
• Offspring were born from frozen SSCs by natural mating
• Offspring showed normal genomic imprinting patterns and gene expression

## Graphical abstract

**Figure:**
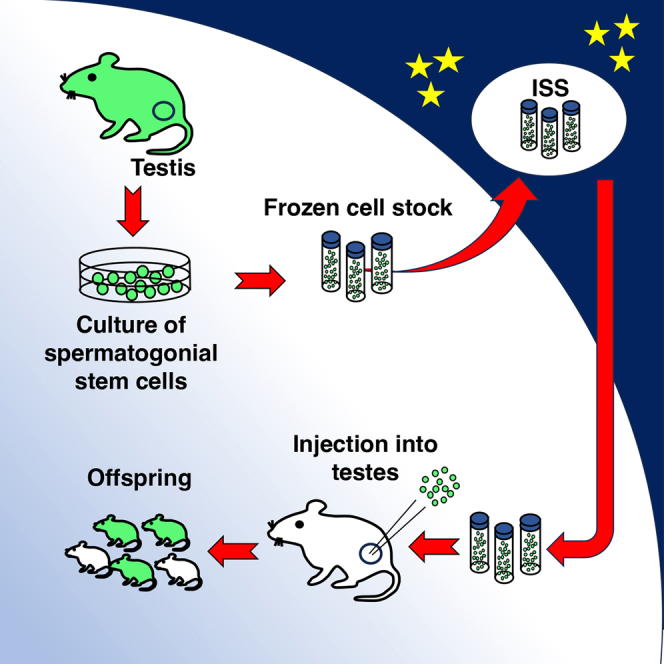

## Teaser

Kanatsu-Shinohara et al. demonstrate that spermatogenesis is possible from SSCs cryopreserved for 6 months aboard the International Space Station. The SSCs showed no apparent *in vitro* abnormalities, and healthy-looking offspring were produced via spermatogonial transplantation and natural mating. These findings lay the groundwork for developing germline preservation strategies during prolonged space missions.

## Introduction

It has been over six decades since the first animals were sent into space. Since then, several species, including humans, have experienced long-term spaceflight and safely returned to Earth. However, during spaceflight, stressors, such as radiation, microgravity, hypergravity, and circadian rhythm disruption are inevitable (Mishra and Luderer, 2019). These hazards can lead to varying degrees of organ dysfunction, including stem cells. Common post-flight effects include muscle wasting and decreased bone mineral density, yet the full biological impact of spaceflight remains incompletely understood. Studying stem cells during and after spaceflight is critical, as damage to these cells can result in long-term tissue dysfunction. While most missions have lasted only several months—the longest being 437 days (Kotovskaya et al., 2019)—the prospect of multi-year deep space missions, planetary colonization, and space tourism is becoming realistic. Therefore, it is essential to understand how spaceflight impacts stem cell biology.

Studying germ cells is particularly important since they directly influence the next generation. Given the long lifespan of humans, detailed studies using animals with shorter lifespans are especially valuable in this context. In early studies of rats, no significant changes in the testes were reported (Portugalov et al., 1976; Plakhuta-Plakutina, 1977). However, Fedrova et al. observed a 30%–70% increase in atypical spermatozoa, such as curled tails or the absence of tails, in dogs after 75 days of spaceflight (Fedrova, 1967). Additionally, a 5%–10% decrease in spermatogonia was noted in rat testes during the COSMOS 2044 and COSMOS 1887 missions (Amann et al., 1985; Sapp et al., 1990). More severe damage was observed in mouse testes after 91 days of spaceflight, with experiments revealing significant testicular degeneration and many tubules almost completely devoid of spermatogenic cells, except for a few remaining spermatogonia (Masini et al., 2012). Similar effects were observed in hindlimb suspension models, which simulate spaceflight conditions and lead to abnormal spermatogenesis (Hargens et al., 1984; Tou et al., 2002). Immobilization of primates also resulted in the arrest of spermatogenesis (Zemjanis et al., 1970). Thus, abnormal spermatogenesis in space likely affects many animal species, including humans.

Due to these dramatic environmental changes, analyzing physiological functions in animals during spaceflight is complicated by both systemic and local factors. For example, in some studies, over half of the experimental mice died due to spaceflight-related stresses (Masini et al., 2012). Some systemic effects are mediated by disruptions in the hypothalamic-pituitary-testis axis. Serum, urine, and salivary testosterone levels significantly decreased during flight compared to pre-flight levels (Mishra and Luderer, 2019; Ahrari et al., 2022). Animals also exhibited increased serum luteinizing hormone levels, consistent with decreased negative feedback from the testes. Interestingly, testosterone levels in serum or intratesticular tissue did not seem to be affected by irradiation (Cunningham and Huckins, 1978), suggesting that spaceflight-induced stress is more severe than radiation alone. In addition to hormonal changes, local testicular cell-cell interactions also affect germ cell survival. Gene expression in Sertoli cells, which are in direct contact with germ cells, is influenced by microgravity (Cirelli et al., 2017). While the risks of spaceflight are well known, most studies have focused on damage to somatic cells, and much remains to be learned about the mechanisms behind abnormal spermatogenesis during spaceflight.

One potential approach to addressing these issues is to examine the function of pure germ cells stored in space. Sperm cells are likely the most resistant cell type in the body, capable of surviving and maintaining fertility even after exposure to 25 Gy (Wakayama et al., 2021). This is in contrast to most somatic cells, which undergo apoptosis when exposed to 1–2 Gy. Leveraging this resilience, Wakayama et al. reported in 2017 that freeze-dried mouse spermatozoa exposed to space ionizing radiation for 288 days on the International Space Station (ISS) showed no significant changes in fertilization or birth rates (Wakayama et al., 2017). Although a slight increase in sperm DNA damage was observed, fertilization was still successful via intracytoplasmic sperm injection (ICSI) into oocytes. These findings are promising and suggest freeze-drying as a potential method for germline conservation. Offspring were successfully produced from freeze-dried sperm stored on the ISS for up to 5 years and 10 months. However, ICSI affects genomic imprinting and may lead to behavioral and morphological abnormalities (Fernández-González et al., 2008; Esteves et al., 2018; Lewon et al., 2020; Kanatsu-Shinohara et al., 2023), and extended analysis is required for understanding the impact of using freeze-drying sperm.

Previous studies report detrimental effects of spaceflight on stem cells. For example, while embryonic stem (ES) cells may serve as an alternative resource for germline preservation, several groups report the upregulation of apoptosis- and senescence-associated genes, and no offspring are born from ES cells stored in space (An et al., 2019; Yoshida et al., 2024; Mao et al., 2024). Specifically, the downregulation of DNA repair genes has raised concerns about potential germline damages. It is possible that embryonic cells are particularly sensitive to spaceflight because preimplantation embryos exhibited significantly poor *in vitro* development during spaceflight, likely due to irradiation (Lei et al., 2020). Another study also reported ectopic expression of an inner cell mass marker in the blastocyst cavity (Wakayama et al., 2023). As a result, currently artificial reproductive technologies may have limited applications for germline preservation in space, raising concerns about reproductive protection during human spaceflight.

Spermatogenesis begins with spermatogonial stem cells (SSCs), which continuously self-renew and produce numerous sperm (Meistrich and van Beek, 1993; de Rooij and Russell, 2000). As they differentiate, SSCs give rise to differentiating spermatogonia, which are particularly sensitive to radiation (De Felice et al., 2019). This high sensitivity may explain the significant loss of spermatogonia observed in mouse testes during spaceflight. One unique characteristic of SSCs is their ability to increase in number through stimulated self-renewal. The addition of glial cell line-derived neurotrophic factor (GDNF) and fibroblast growth factor (FGF)2, both key factors in SSC self-renewal, allows for the *in vitro* expansion of cultured SSCs, designated as germline stem (GS) cells, for over 2 years (Kanatsu-Shinohara et al., 2003a; Kanatsu-Shinohara and Shinohara, 2013). This provides a new opportunity, as SSCs, unlike sperm, undergo meiosis, providing a unique resource for generating genetic diversity. Another advantage of SSCs is their straightforward freezing protocol (Avarbock et al., 1996). In contrast, sperm cryopreservation requires species-specific protocols due to the unique head morphology of sperm (Holt, 2000), making it difficult to establish a universal method. However, SSCs from various species, including humans, can be frozen similarly to somatic cells. Offspring have been produced by transplantation of cryopreserved SSCs by natural mating (Kanatsu-Shinohara et al., 2003b).

In this study, we utilized SSCs for long-term cryopreservation during spaceflight. GS cells were frozen for 6 months aboard the ISS. Although live animals have been maintained on the ISS for up to 3 months (Cancedda et al., 2012), the analysis of germ cells in these animals remains limited due to the challenges of long-term maintenance of live animals in space. Since frozen cells lack DNA repair mechanisms, they offer a sensitive experimental system to study potential damage in SSCs. Upon return to Earth, the frozen SSCs were thawed, and their DNA damage and gene expression profiles were analyzed. The impact on SSC activity was further evaluated through spermatogonial transplantation into infertile mouse testes.

## Results

### Survival of SSCs after cryopreservation and irradiation on Earth

**Figure 1:**
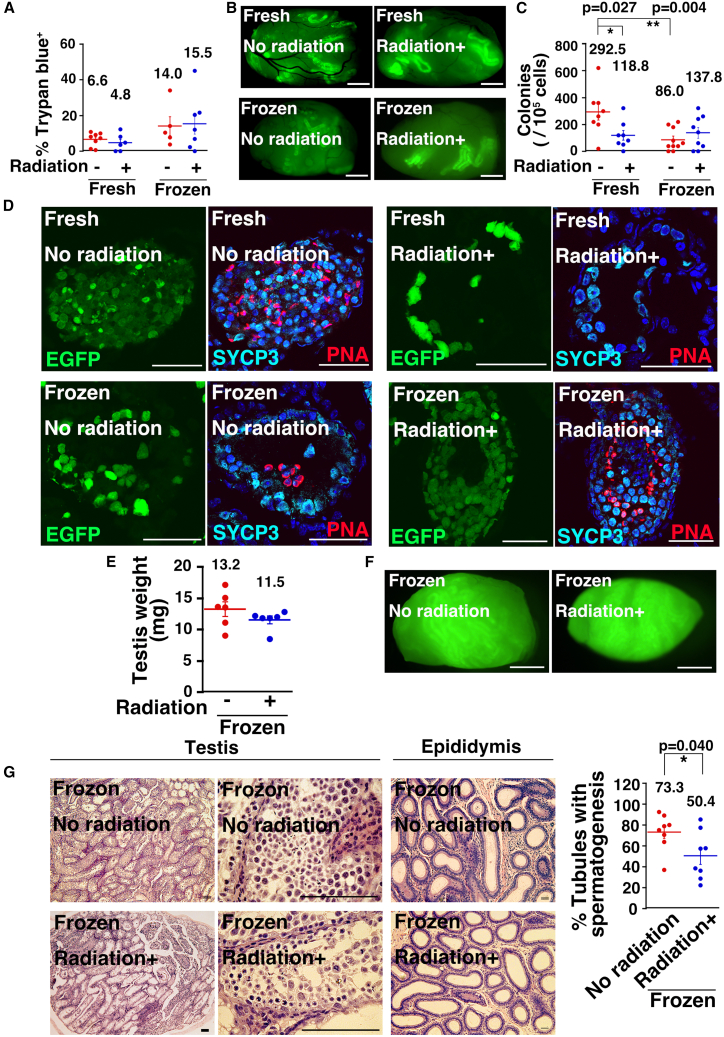
Impact of radiation on frozen GS cells (A) Cell survival rate after freezing and radiation (*n* = 8, 6, 5, and 7 experiments for fresh, fresh radiation, frozen, and frozen radiation, respectively). (B) Macroscopic appearance of busulfan-treated recipient testis transplanted with GS cells. (C) Colony counts (*n* = 8, 8, 10, and 9 testes for fresh, fresh radiation, frozen, and frozen radiation, respectively). (D) Immunostaining of recipient testis for SYCP3 (spermatocyte marker) and PNA (haploid cell marker). (E) Testis weight (*n* = 6 testes). (F) Macroscopic appearance of W recipient testis transplanted with GS cells. (G) Histological appearance of recipient testis and epididymis. 824 and 874 tubules were counted in 8 testes for no radiation and radiation, respectively. Stain, hematoxylin and eosin (G) and Hoechst 33342 (D). Bar = 1 mm (B and F), 50 μm (D), and 100 μm (G). Each bar represents the mean ± SEM, and the numerical mean value is indicated above each bar. ^∗^*p* < 0.05; ^∗∗^*p* < 0.01.

Since cryopreservation inhibits the DNA repair mechanisms, we first assessed the effects of cryopreservation and irradiation on SSCs on Earth. Four types of GS cells were used: fresh non-irradiated, fresh irradiated, frozen non-irradiated, and frozen irradiated GS cells. Irradiation involved exposing dissociated GS cells to 1.5 Gy, the median lethal dose 50 for GS cells (Ishii et al., 2014a; 2014b). Cryopreservation was performed in a DMSO-based solution. To evaluate cell viability, we used trypan blue staining, which showed that both frozen groups had a higher proportion of dead cells compared to fresh groups (Figure 1A), but no significant differences were found among the four samples.

Two experiments were conducted using spermatogonial transplantation to further evaluate SSCs (Brinster and Zimmermann, 1994). In the first experiment, SSC frequency was determined by counting colonies in busulfan-treated mice, which have empty tubules. Two months after transplantation into busulfan-treated mice, the mice were sacrificed, and the number of colonies was counted (Figure 1B). Colony numbers were 292.5, 118.8, 86.0, and 137.8 per 10^5^ transplanted cells, for fresh, fresh irradiated, frozen, and frozen irradiated cells, respectively (Figure 1C). While survival rates were comparable, fresh cell transplantation produced significantly more colonies, and irradiation reduced the number of colonies to 40.6% in fresh GS cells, while frozen cells showed a 60.2% increase in colony formation post-irradiation. However, there was no statistical significance. Immunostaining of recipient testes showed that irradiated fresh cells produced very few tubules with peanut agglutinin (PNA)^+^ haploid cells, though these tubules contained SYCP3^+^ meiotic spermatocytes (Figure 1D).

In the second set of experiments, we aimed to confirm the extent of complete differentiation, as this information is critical for producing offspring through natural mating. We used congenitally infertile WBB6F1-W/W^v^ (designated W) mice and compared irradiated frozen GS cells with non-irradiated frozen cells. Unlike busulfan-treated mice, W mice lack endogenous spermatogenesis due to mutations in the *Kit* tyrosine kinase receptor (Fleischman, 1993). By transplanting a large number of GS cells, we were able to evaluate the donor cells’ complete differentiation capacity. Three months post-transplantation, there was no difference in testis weight between the two groups (Figure 1E). Due to the higher number of transplanted cells (4 × 10^5^ cells per testis), donor cell differentiation enhanced in the recipient testes, as indicated by long green segments of seminiferous tubules under UV light (Figure 1F). Histological analysis showed complete spermatogenesis in both groups (Figure 1G). However, the number of tubules undergoing spermatogenesis was lower following transplantation of irradiated frozen GS cells compared to non-irradiated GS cells. Given that the number of germ cell colonies did not differ significantly (Figure 1C), this result suggests that regeneration is delayed by irradiation. Nevertheless, the presence of spermatozoa in the epididymides of all recipients indicates that they may still be capable of achieving fertility through natural mating.

### Evaluation of total space radiation

For storage on the ISS, GS cells were cryopreserved and stored in the Minus Eighty-Degree Laboratory Freezer (MELFI) freezer for 6 months. A control set of GS cells was also cryopreserved and stored in the MELFI freezer on Earth. The samples were launched to the ISS on July 14, 2022, and returned to Earth on January 11, 2023, remaining in space for a total of 181 days. The cells were kept at −95°C during the spaceflight and at −80°C during transport on Earth. Upon arrival in Kyoto, the cells were stored in liquid nitrogen for subsequent analysis.

**Figure 2:**
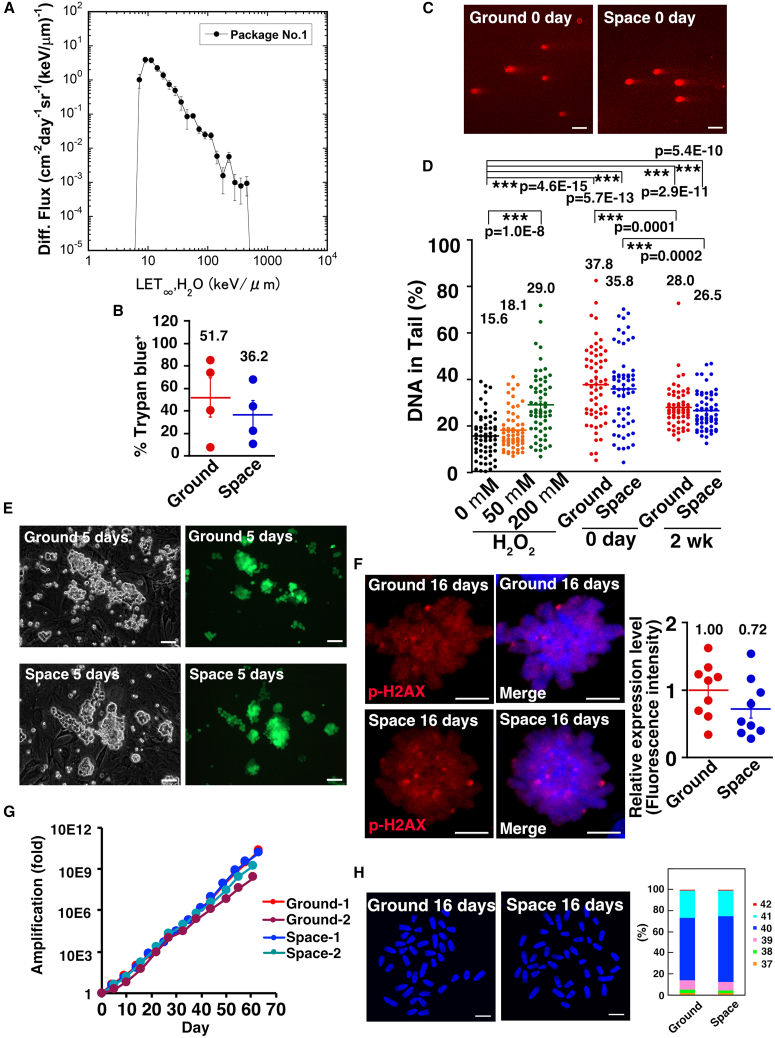
Phenotype of Space GS cells (A) Differential particle flux function of LET, H_2_O measured with CR-39 PNTDs in flight package no. 1 measured by PADLES. This LET distribution was obtained by subtracting the background LET distribution measure with the CR-39 PNTDs in TKSC ground control package no. 2. (B) Viability of GS cells by trypan blue staining, immediately after thawing (*n* = 4 experiments). (C and D) Comet assay (C) and quantification (D) on the day of thawing. The head (round shape) corresponds to undamaged DNA, while the tail (smear) corresponds to damaged DNA. Sixty cells were picked up, and the intensity ratio of head over total DNA was quantified. (E) Appearance of Space GS cells *in vitro* 5 days after thawing. (F) Immunostaining of Space GS cells 16 days after thawing with two rounds of passages. Nine cells at metaphase stage were analyzed for p-H2AX expression level. (G) Proliferation of Space GS cells. (H) Karyotype of Space GS cells 16 days after thawing with two rounds of passages. One hundred cells were analyzed for counting chromosome number. Bar = 50 μm (C and E) and 5 μm (F and H). Each bar represents the mean ± SEM, and the numerical mean value is indicated above each bar. ^∗∗∗^*p* < 0.001.

To evaluate the radiation dose, we analyzed the bio passive dosimeter for life science experiments in space (PADLES) (Figure 2A), which contains CR-39 plastic nuclear track detector (PNTD) for measuring radiation dose and characteristics (Nagamatsu et al., 2013). PADLES was placed near the frozen samples to accurately measure the cosmic radiation dose. Analysis of the PADLES surface revealed etch marks from nuclear tracks created by atomic nuclei during spaceflight. The linear energy transfer (LET) distribution was determined by subtracting the background LET distribution measured with PNTDs from the ground control package at the Tsukuba Space Center (TKSC). According to PADLES, the absorbed radiation dose rate was 0.31 ± 0.01 mGy per day, corresponding to a dose-equivalent rate of 0.59 ± 0.03 mSv per day. The total absorbed dose was 106.5 ± 5.2 mSv.

We thawed the cells and assessed survival using trypan blue staining, with approximately 50%–60% of the cells surviving post-thaw (Figure 2B). No significant differences were observed between GS cells stored in space (Space GS cells) and those on Earth (Ground GS cells) (Figure 2B). We also conducted an alkali comet assay to detect both single-strand and double-strand DNA breaks (Figure 2C). As a positive control, we treated GS cells with hydrogen peroxide at two different doses (50 and 200 μM) for 30 min.

Unexpectedly, analysis of Space GS cells did not show an increase in DNA damage compared to Ground GS cells. However, both Space and Ground GS cells exhibited significantly more DNA damage than fresh GS cells cultured without hydrogen peroxide. Because Space GS cells have undergone exposure to damages by spaceflight, this result suggested that the damage by spaceflight was limited compared to freeze-thawing process. When reanalyzed 2 weeks after thawing and two rounds of passages, both types of cells showed significantly reduced DNA damage compared to freshly thawed cells. Nevertheless, the damage was not completely repaired, as more DNA damage was present than in fresh GS cells. Both types of cells exhibited noticeable DNA damage upon thawing, but no difference was found in the intensity ratio of head over total DNA between the two samples (Figures 2C and 2D).

When Space GS cells were cultured *in vitro*, their colony morphology was indistinguishable from Ground GS cells (Figure 2E), showing grape-like clusters of spermatogonia. Immunostaining of cells cultured for 16 days after thawing and two passages revealed that the expression levels of phosphorylated-H2AX, a marker of DNA damage, were comparable between Ground and Space GS cells (Figure 2F). These cells proliferated normally for at least 100 days. The proliferation rate was comparable between Space and Ground GS cells, with doubling times of 2.0 and 1.8 days, respectively (Figure 2G). Since an abnormal karyotype can impair spermatogenesis and cause infertility, we also evaluated the karyotype of Space GS cells 16 days after thawing with two rounds of passages, revealing that 63% of the cells had 40 chromosomes (Figure 2H). The difference was not statistically significant.

### Gene expression and DNA methylation in Space GS cells

**Figure 3:**
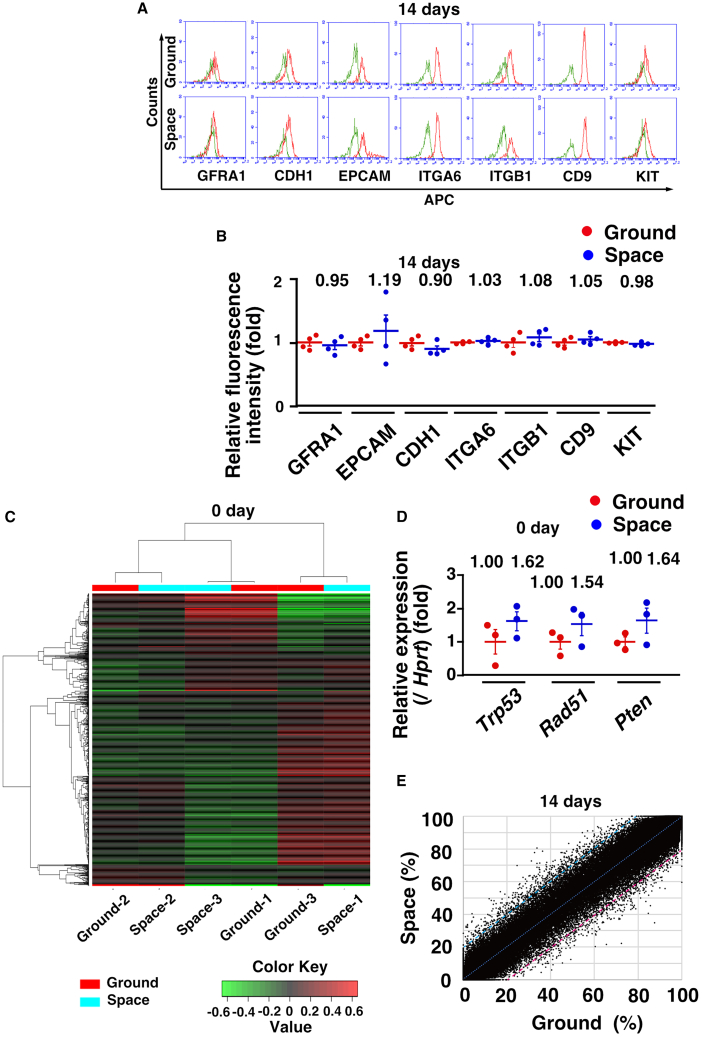
Analysis of Space GS cells (A) Flow cytometric analysis using antibodies against spermatogonia markers. Green lines indicate controls. The cells were analyzed 14 days after thawing with two rounds of passages. (B) Mean fluorescence intensity (*n* = 4 experiments). (C) Heatmap of RNA-seq. The cells were analyzed on the day after thawing without passages. (D) Real-time PCR analysis immediately after thawing (*n* = 3). (E) A scatterplot showing correlation of the DNA methylation data at individual CpG sites in gene promoters (*n* = 3 experiments). The numbers of identified hypermethylated sites and hypomethylated sites in Space and Ground GS cells are shown in red and blue, respectively, along with the percentages of commonly covered sites. Red or blue lines indicate 20% increased methylation levels or 20% decreased methylation levels in Space GS cells, respectively. The dashed line indicates the linear regression line. The cells were analyzed 14 days after thawing with two rounds of passages. Each bar represents the mean ± SEM, and the numerical mean value is indicated above each bar. See also Figure S1.

To assess the phenotype of Space GS cells, we first performed flow cytometry with antibodies against spermatogonia surface markers. A comparison between Space and Ground GS cells showed no significant differences in the mean fluorescence intensity for the tested antigens (Figures 3A and 3B). Considering previous reports on ES cells that indicated downregulation of DNA repair genes and upregulation of senescence- and apoptosis-associated genes (An et al., 2019; Mao et al., 2024), we conducted RNA sequencing (RNA-seq) to identify genes specifically induced by spaceflight. Using three lines of Space and Ground GS cells, we performed hierarchical clustering analysis, but no significant differences in gene expression profiles were observed (Figure 3C; Table S1). Real-time PCR analyses showed no significant changes in the expression levels of *Trp53*, *Rad51*, and *Pten* (Figure 3D).

Additionally, we conducted reduced representation bisulfite sequencing (RRBS) to examine potential changes in DNA methylation patterns (Figures 3E and S1). Among 681,804 covered CpG sites covered in gene promoters, our analysis identified 770 hypermethylated sites (0.113% of commonly covered sites) and 563 hypomethylated sites (0.083% of commonly covered sites) in Space GS and Ground GS cells, respectively (>20% change, R^2^ = 0.9912; Table S2). There were no significant differences in the overall DNA methylation of promoter CpG sites between Space GS cells and Ground GS cells (Figure S1). Only four genes were differentially methylated between the two groups: *Gm7097*, *Bsnd*, and *Fgf21* were hypermethylated in Space GS cells, while *Gm15562* was hypomethylated in ground GS cells. *Fgf21* was downregulated in Space GS cells, while *Gm15562* was expressed only in Ground GS cells according to RNA-seq data (Table S3). The two other genes were not expressed in either type of GS cells. No significant differences in DNA methylation patterns were observed in imprinted genes. These findings suggest that the phenotype of GS cells did not change significantly after spaceflight.

### Functional analysis of space GS cells by spermatogonial transplantation

**Figure 4:**
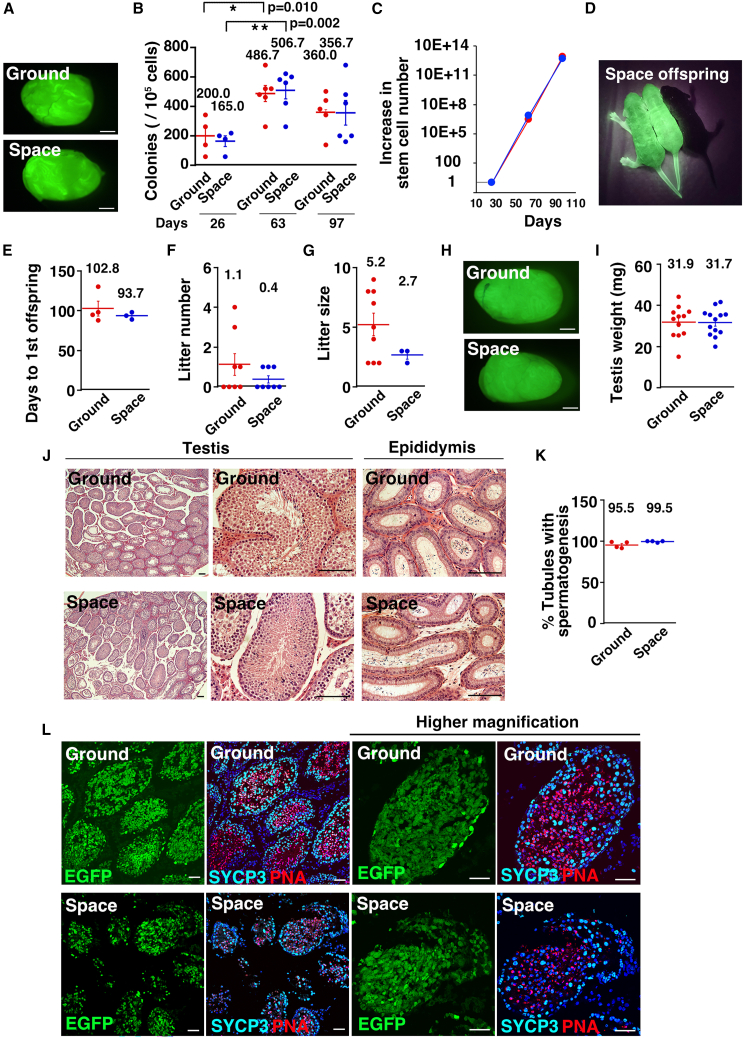
Functional analysis of Space GS cells (A) Macroscopic appearance of adult W testis transplanted with Space GS cells. (B) Colony count (*n* = 4, 6, and 5 [ground] and 4, 6, and 6 [space] testes for 26, 63, and 97 days, respectively). (C) Increase in SSC number from the first transplantation. To evaluate the total stem cell number during culture, we transplanted a portion of cells at the time of passages into infertile mice. The total number of SSCs was estimated by multiplying the total cell counts by the stem cell concentration, as determined by transplantation assay. (D) Offspring born from W recipient transplanted with Space GS cells, showing donor cell-derived green fluorescence. (E) Days to first offspring (*n* = 4 mice for Ground; *n* = 3 mice for Space). (F) Litter number (*n* = 8 experiments). (G) Litter size (*n* = 9 experiments for Ground; *n* = 3 experiments for Space). (H) Macroscopic appearance of pup W testis transplanted with Space GS cells. (I) Testis weight (*n* = 12 testes for Ground, *n* = 13 testes for Space). (J) Histological appearance of testis and epididymis. Epididymides were from infertile recipients. (K) Frequencies of tubules with spermatogenesis. 402 and 352 tubules in 4 testes were counted for Ground and Space GS cell recipients, respectively. (L) Immunostaining of W mouse testis for SYCP3 (spermatocyte marker) and PNA (haploid cell marker). Bar = 1 mm (A and H), 100 μm (J), and 50 μm (L). Each bar represents the mean ± SEM, and the numerical mean value is indicated above each bar. ^∗^*p* < 0.05; ^∗∗^*p* < 0.01. See also Figure S2.

Since only a portion of GS cell contain SSCs (Kanatsu-Shinohara and Shinohara, 2013), we performed spermatogonial transplantation experiments during 3 months of culture. Both types of cells proliferated at a comparable rate, with no significant changes in growth characteristics. The cells were transplanted into the seminiferous tubules of busulfan-treated mice at 26, 63, and 97 days after thawing. When the number of colonies was counted 2 months post-transplantation, we observed significant increases in the number of germ cell colonies at 63 and 97 days compared to 26 days (Figures 4A and 4B). Although no significant difference between Ground GS cells and Space GS cells was noted at 26 days, both exhibited poor colonization levels compared to longer-term cultures. However, these values were within the previously reported results, which ranged from 2.0 to 800.0 per 10^5^ cells (Kanatsu-Shinohara et al., 2005; Miyazaki et al., 2023). SSCs increased logarithmically at comparable rates (Figure 4C; Table S4). These results suggest that spaceflight did not affect the balance between SSCs and progenitors *in vitro*.

Based on these observations, we next examined the feasibility of producing offspring from Space GS cells. Immature W pups were used as recipients, as donor cells colonized these recipients 5 to 10 times more efficiently than adult W mice (Shinohara et al., 2001). The absence of a blood-testis barrier in immature testes is believed to enhance SSC homing to germline niches (Kanatsu-Shinohara et al., 2020). After transplantation, the pups were returned to their mothers for sexual maturation. At about 1 month of age, the recipient mice were housed with wild-type C57BL/6 (B6) females. One of the offspring became fertile as early as 89 days after transplantation. Although no donor fluorescence was observed in the first litter, EGFP^+^ offspring were found in the second litter, confirming the donor cell origin of the offspring (Figure 4D; Space pups). Overall, 3 of 8 mice became fertile from Space GS cells. Offspring were also obtained from Ground GS cells (Ground pups) as early as 88 days after transplantation, with 4 of 8 recipients becoming fertile. The number of days to the first offspring, the total number of litters, and the litter size did not show significant differences during ∼5 months of natural mating (Figures 4E–4G).

Six months after transplantation, the mice were sacrificed, and their testes were analyzed (Figure 4H). No significant differences were observed in testis size between the two groups (Figure 4I). Histological examination of the testes revealed normal spermatogenesis in both groups (Figure 4J). Quantification of seminiferous tubules with spermatogenesis showed no significant differences between Space and Ground GS cells (Figure 4K). While sperm were present in the epididymides of fertile recipients, they were similarly found in 50% of the epididymides of infertile recipients (Figure 4J). Immunostaining of recipient testes confirmed the presence of SYCP3^+^ spermatocytes and PNA^+^ haploid cells, with no morphological abnormalities (Figure 4L). These results suggest that transplantation of a larger number of cells might have allowed offspring production.

### Analysis of the offspring

Given that ICSI can cause abnormalities in genomic imprinting (de Waal et al., 2012), we used combined bisulfite restriction analysis (COBRA) to examine the genomic imprinting of the offspring using tail DNA. Differentially methylated regions (DMRs) of the *H19* and *Igf2r* genes were analyzed. All offspring exhibited normal somatic DNA methylation patterns, with no apparent differences between the two types of donor cells (Figure S2A). Bisulfite sequencing further confirmed no abnormalities in the DMRs of these genes (Figure S2B). However, because abnormalities may not be evident by DNA methylation, RNA-seq analysis of liver tissue was performed to verify normal gene expression patterns in Space pups, Ground pups, and wild-type mice born through natural mating. Three F1 males and females from each group were analyzed and compared with three wild-type mice of the same genetic background (Table S5). No significant differences in gene expression were observed among the groups (Figure S2C). These findings suggest that Space GS cells retain fertility and produce normal offspring.

## Discussion

This study has two primary objectives. The first is to evaluate the utility of SSCs for germline preservation in space. Although freeze-dried sperm has been successfully stored and used during spaceflight (Wakayama et al., 2017, 2021), ICSI and freeze-drying procedure may pose long-term potential health risks to offspring. The second objective is to assess potential damage to SSCs caused by cosmic radiation. Spermatogenesis is significantly impaired post-spaceflight, including a reduction in the number of spermatogonia (Mishra and Luderer, 2019), suggesting that SSCs could be particularly sensitive to cosmic radiation. Unlike differentiated germ cells, which differentiate and disappear, any damage to SSCs could persist long-term and accumulate in the germline. Thus, preventing genomic damages in SSCs is crucial for safe spaceflight. In this context, evaluating the fertility of frozen SSCs not only serves as a new biological resource for germline preservation but also helps assess spaceflight-related risks.

Our initial Earth-based experiments showed that frozen GS cells are relatively resistant to irradiation. While DNA repair mechanisms do not function in frozen cells, it has been previously suggested that frozen cells are better protected from irradiation than fresh cells (Aschwood-Smith and Friedmann, 1979; Cugia et al., 2011). These studies reveled a 3.5- to 20-fold higher survival rate for frozen cells stored in liquid nitrogen compared to fresh cells. This observation aligns with finding of fewer gammaH2AX foci (a marker of double-strand DNA breaks) in frozen irradiated cells compared to fresh irradiated cells. Although the exact reason for this improved survival is unclear, it has been proposed that the production and diffusion of reactive oxygen species (ROS), which can damage DNA and cell structures, are reduced in the frozen state (Cugia et al., 2011). In our study, however, we did not observe improved survival rates in irradiated frozen GS cells. This may be due to the storage conditions: ideally, cells should be stored at −196°C in liquid nitrogen, whereas we used a deep freezer due to the unavailability of liquid nitrogen on the ISS. Nevertheless, the relatively high resistance of frozen GS cells to irradiation formed the basis for our space-based experiments.

Our analysis of Space GS cells revealed no significant increase in damage using the comet assay. Given the notable damage observed in ES cells and early embryos exposed to spaceflight (An et al., 2019; Lei et al., 2020; Wakayama et al., 2023), we expected GS cells would show heightened apoptosis, as they are more vulnerable to DNA double-strand breaks compared to ES cells (Ishii et al., 2014a, 2014b). This is not surprising because ES cells are notorious for their high sensitivity to radiation (Aladjem et al., 1998; Hong and Stambrook, 2004). Contrary to these expectation, the comet assay showed that cryopreservation caused more damage than spaceflight in GS cells. Hydrogen peroxide concentration used (50 μM) was sufficient to kill both mouse embryonic fibroblasts and ES cells in a week (Mori et al., 2021), yet only minimal differences were observed between Space GS cells and Ground GS cells, suggesting that cells in the cryopreserved state have some specific protective mechanism against radiation. Although radiosensitivity in ES cells can be improved by overexpression of *Chk2* gene by restoring a G1 arrest (Hong and Stambrook, 2004), this may not apply to GS cells because GS cells have a long G1 phase (Ishii et al., 2014a, 2014b). Instead, since GS cells possess strong base excision repair (BER) activity (Mori et al., 2021), this may have helped protect them from spaceflight-induced damage, differentiating them from ES cells, which have weaker BER activity (Mori et al., 2021).

Further analysis revealed that GS cells proliferated normally *in vitro*. After spermatogonial transplantation, similar numbers of germ cell colonies were observed between Space GS cells and Ground GS cells. Though smaller numbers of colonies were formed 26 days post-culture initiation, this could reflect freeze-thaw damage as indicated by the comet assay (Figure 2C). Despite this initial difference, the number of colonies remained constant, and their morphology showed no substantial differences between the two groups. Over 3 months, there were no significant changes in SSC numbers, indicating that cosmic rays did not disrupt the balance between self-renewal and differentiating in SSCs. Therefore, spaceflight did not impair the SSCs’ ability to recolonize the seminiferous tubules. These results contrast with studies on neural stem cells (NSCs), which showed an 8-fold increase in cell-cycle activity after returning to Earth following space exposure, which normalized after 30 h of re-exposure to 1*g* condition (Shaka et al., 2022). These NSCs demonstrated “memory” effects post-spaceflight, experiencing abnormal cell division patterns, including incomplete or muti-daughter cell divisions. Additionally, spaceflight downregulated the GDNF signaling pathway in ES cells. Because GDNF is an essential self-renewal factor for SSCs, this raised concerns that GS cells may have difficulty responding to self-renewal signals upon thawing (An et al., 2019). However, the normal proliferation of GS cells after spaceflight suggests that self-renewal regulation resumed without issues.

The most notable result of this study was the successful production of offspring through natural mating. These offspring were born within 3–4 months after spermatogonial transplantation, consistent with the expected timeline for fertility restoration following such transplants (Shinohara et al., 2001; Kanatsu-Shinohara et al., 2016). Importantly, the offspring from Space GS cells showed no noticeable congenital defects, normal genomic imprinting patterns, and no significant differences in gene expression. Further analyses, such as lifespan assessments and fertility testing, are required to rule out long-term health issues. In particular, although offspring produced by ICSI did not show any abnormal DNA methylation patterns, additional analyses are required to confirm the long-term health of the offspring as well as their descendants. Nevertheless, the birth of normal-appearing offspring underscores the potential utility of SSCs for germline conservation.

At least two questions arise from the present study. First, it will be crucial to determine how long SSCs can be kept frozen on the ISS. Since the cells are in a frozen state, potential damage from cosmic ray will accumulate without DNA repair mechanisms, potentially compromising SSC function. Although the differences were not statistically significant, analysis of *Trp53*, *Rad51*, and *Pten* mRNA levels revealed increased expression in Space GS cells (Figure 3D). Extended storage on the ISS may lead to greater damage and compromise their reproductive potential. Such damage to SSCs could pose a critical concern for human spaceflight, as human spermatogenesis is generally more sensitive to irradiation than that of mice (Clifton and Brenner, 1983). Second, assessing the impact of genomic damages on the offspring and subsequent generations is essential. Although apparently healthy offspring were born, it is unclear whether spaceflight-induced DNA damages were fully repaired. Previous studies have shown elevated hepatic expression of DNA replication-related genes in offspring produced via *in vitro* fertilization using sperm from space-exposed males (Yoshida et al., 2021). Additionally, mice produced via ICSI using freeze-dried sperm after spaceflight had a shorter lifespan than typical B6 mice (604 days vs. 901 days) (Wakayama et al., 2021). Long-term observation is crucial to understand the effects of prolonged cosmic ray exposure.

In conclusion, cosmic influences on stem cells are a critical issue for long-term human space habituation. The successful production of offspring from cryopreserved SSCs demonstrates the utility of SSCs for germline preservation in space. By using spermatogonial transplantation, it is possible to distinguish the effects of spaceflight on germ cells and somatic cells. However, while healthy offspring were obtained, much remains to be explored regarding the risks of spaceflight on SSCs. Given the long reproductive lifespan of humans, animal models with shorter lifespans are essential for such research. As the space industry continues to expand, these experiments are necessary prerequisites for future long-term human spaceflights. The next logical step would be to culture GS cells on the ISS and monitor the impact of cosmic radiation on live germ cells, which may provide further insights into DNA repair mechanisms and enhance the technique’s applicability for space missions. Thus, our successful production of offspring from cryopreserved SSCs offers a solid foundation for future space-based applications.

## Methods

### Animals and transplantation procedure

For colony counting, cells were microinjected into the seminiferous tubules of mature B6 × DBA/2 F1 (BDF1) mice (Japan SLC, Shizuoka, Japan). These mice received intraperitoneal injection of busulfan (44 mg/kg; Sigma, St. Louis, MO) to remove endogenous spermatogenesis at 4 weeks of age and were used at least 1 month after busulfan treatment. For assessment of spermatogenesis differentiation levels, 4- to 8-week-old W mice were used. For fertility experiments, we used 5- to 10-days-old W pups (Japan SLC), as described previously (Shinohara et al., 2001). All injection was performed via efferent duct, and 75%–85% of the tubules were filled with the cell suspension, as described previously (Ogawa et al., 1997). Approximately 10 μL was microinjected into BDF1 testis, whereas 2 μL was injected into W pups because they were smaller. The Institutional Animal Care and Use Committee of Kyoto University approved all animal experimentation protocols.

### COBRA

Genomic DNA was treated with sodium bisulfite, which deaminates unmethylated cytosines to uracils but does not affect 5-methylated cytosines. Bisulfite-treated DNA was used as a template for amplifying DMRs using specific primers. The amplified DNA was cut with methylation-sensitive restriction enzymes, as described previously (Kanatsu-Shinohara et al., 2004). The PCR primers used in the experiments are shown in Table S7.

### Bisulfite sequencing

DNA methylation analysis was performed as described previously (Kanatsu-Shinohara et al., 2023). Bisulfite-treated DNA was amplified by PCR using specific primer sets, and the amplified PCR products were purified using FastGene gel/PCR extraction kit (NIPPON Genetics, Tokyo, Japan) according to the manufacturer’s protocols. DNA methylation analyses were performed using the Quantification Tool for Methylation Analysis (QUMA) (http://quma.cdb.riken.jp/top/quma_main_j.html). The PCR primers used are listed in Table S7.

### Statistical analyses

Significant differences between means for single comparisons were determined by Student’s t test. Karyotype abnormalities were analyzed by chi-squared test. Significant difference was indicated by the asterisks, as follows: ^∗^*p* < 0.05; ^∗∗^*p* < 0.01; ^∗∗∗^*p* < 0.001.

## Resource availability

### Lead contact

Requests for further information and resources should be directed to and will be fulfilled by the lead contact, Takashi Shinohara (tshinoha@virus.kyoto-u.ac.jp).

### Materials availability

This study did not generate new reagents.

### Data and code availability

RNA-seq and RRBS data have been deposited in the Gene Expression Omnibus and accessible through accession numbers GSE280403, GSE280404, and GSE279277.

## Acknowledgments

This research utilized the Japanese Experiment Module of the International Space Station with the support of the JAXA. Financial support for this research was provided by the 10.13039/501100004020JAXA, 10.13039/100009619AMED (JP24zf0127011), and 10.13039/501100001700MEXT (25H01357, 24H02045, 24K21291, 23K27727, 23H00399, and 23K20043). We thank Dr. Akira Higashibara, Mr. Tetsuya Sakashita, Mr. Yasuhiro Nakamura, Dr. Kazumi Koga, Mr. Yuho Amagai, Dr. Toru Shimadzu, Ms. Hiromi Sano, Ms. Ikuko Osada, Ms. Suzuki Tomoko, Mr. Daisuke Kawamura, Mr. Keisuke Aida, Mr. Hosogai Aki, and JAXA Flight Control Team and Payload Flight Control Team for help with performing the current study.

## Declaration of interests

The authors declare no competing interests.

## Supplemental information

Data S1. Tables S1–S7

Document S2. Article plus supplemental information
