# Supplementary material for: Germline transmission of cryopreserved mouse spermatogonial stem cells maintained on the International Space Station
Source: Stem Cell Reports. 2025 Aug 14;20(9):102602. doi: 10.1016/j.stemcr.2025.102602 (PMC12447337; doi:10.1016/j.stemcr.2025.102602)
Supplement: Document S1. Figures S1 and S2 and supplemental methods [file mmc1.pdf]

Stem Cell Reports, Volume *20*

Supplemental Information

Germline transmission of cryopreserved mouse spermatogonial stem cells maintained on the International Space Station

Mito Kanatsu-Shinohara, Takuya Yamamoto, Yusuke Shiromoto, Hiroko Morimoto, Tianjiao Liu, Tohru Yamamori, Tomokazu Yamasaki, and Takashi Shinohara

Figure S1

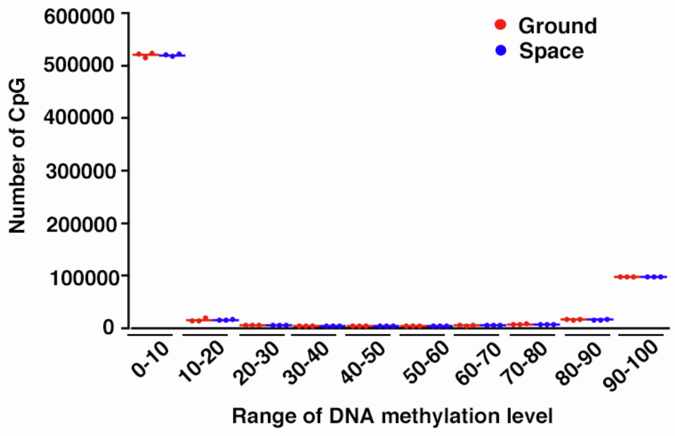

Figure S1. A histogram showing the number of CpG sites at any given DNA methylation level. The number of CpG sites was evaluated using 10% intervals for the rage of 0 to 100%. All promoter methylation sites were included in the determination of the mean of triplicate samples with a minimum read coverage of 10.

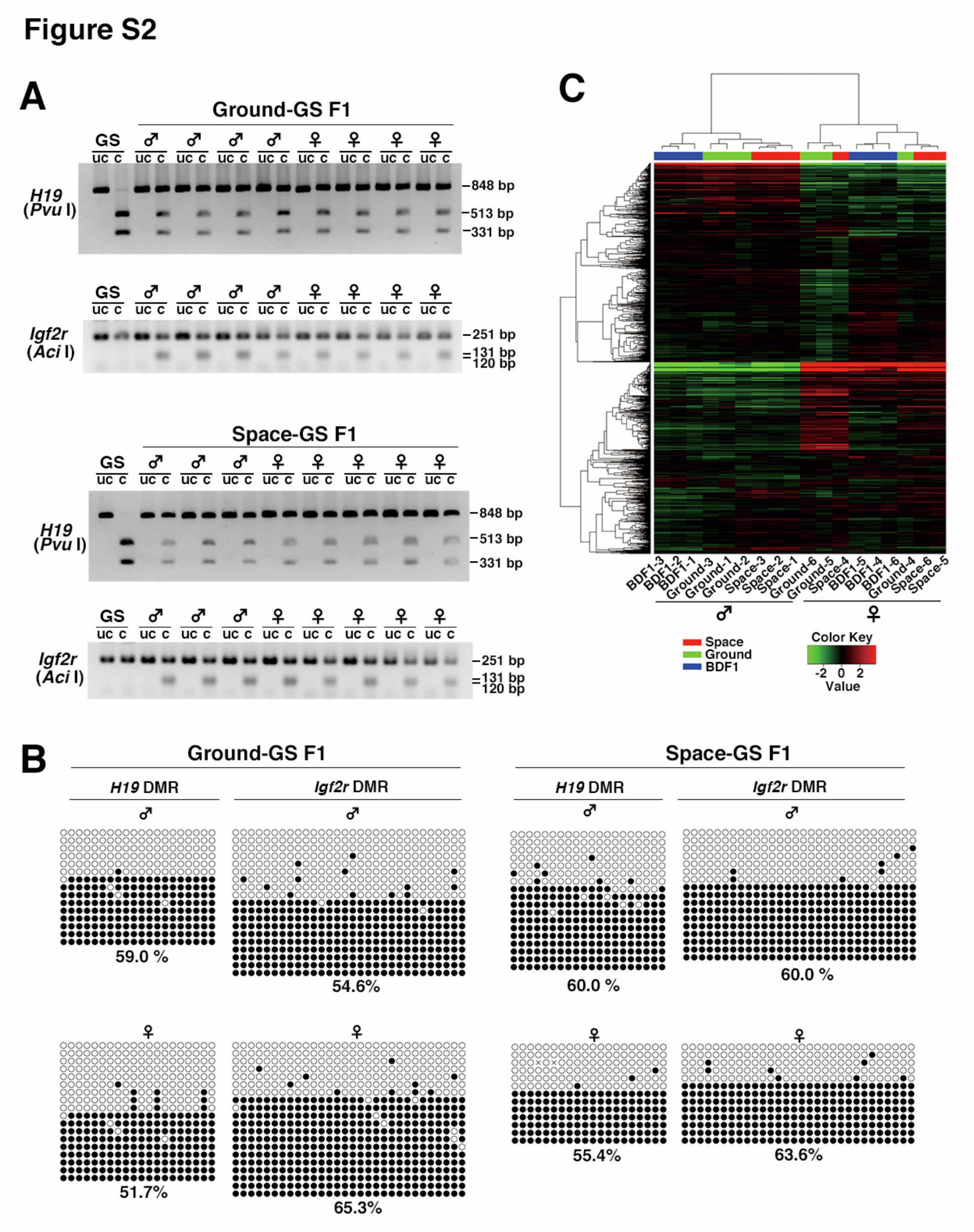

Figure S2. Analysis of genomic imprinting and gene expression in Space pups. (A) COBRA of tail DNA. (B) Bisulfite sequencing of tail DNA. (C) Heatmap of the top 1,000 genes with the most variable read counts in the RNAseq data of liver tissues.

Supplemental Methods

Culture conditions

GS cells used in the present study were previously described (Kanatsu-Shinohara et al., 2003). These cells were derived from C57BL6/Tg14(act-EGFP-OsbY01) mice that was bred into DBA/2 background (Okabe et al., 1997). The culture medium for GS cells included GDNF and FGF2, and cells were cultured on MEFs with 1% fetal bovine serum (Kanatsu-Shinohara et al., 2014). For cryopreservation, 1×10^6 GS cells were suspended in a cryopreservation solution (1ml; Cellbanker; DIA-IATRON, Tokyo, Japan). After pacing the cryotube (Nunc Biobanking and Cell Culture Cryogenic Tube) in a freezing container (CryoBoxes)(both from Thermo Fisher, MA), cells were frozen at -80°C. These tubes and containers were used in a previous study for ES cells (Yoshida et al., 2024). The samples and PADLES were launched to the ISS by SpaceX CRS-25 (SpX-25) on July 14, 2022, maintaining their frozen status and stored in MELFI at -95°C. After approximately 6 months, the samples were returned to the ground by Spade X CRS-26 (SpX-26) on January 11, 2023, maintaining their frozen status. At the ISS, the frozen vials were kept in MELFI. For thawing, the cryotubes were immersed in a water-bath at 37°C, and 10 ml of culture medium was added drop-wise. After washing by centrifugation, the cells were suspended in culture medium. For irradiation experiments, cells were frozen for 3 to 5 days.

RNA-seq

RNA-seq was carried out, as previously described (Kanatsu-Shinohara et al.,

2023). In brief, total RNA was purified using the RNeasy Plus Mini Kit (Qiagen,Valencia, CA) and cDNA libraries were generated using a TruSeq stranded mRNA library preparation kit (Illumina, San Diego, CA). Sequencing was performed using NextSeq550 (Illumina) with a single-read sequencing length of 76 bp (Kanatsu-Shinohara et al., 2023). The sequenced reads were mapped to the mm10 mouse reference genome using HISAT2, with the GENCODE M21 annotation gtf file after trimming adaptor sequences and low-quality bases with cutadapt-1.16 (Martin et al., 2011). Gene ontology analysis was performed using iDEP96 tool (http://bioinformatics.sdstate.edu/idep96/).

Immunostaining

Testis samples were fixed in 4% paraformaldehyde for 2 h at 4°C, embedded in Tissue-Tek OCT compound (Sakura Finetek, Tokyo, Japan) and used for cryosectioning. To block non-specific antibodies, sections were treated with 3% bovine serum albumin (BSA) and 10% donkey serum in phosphate-buffered saline (PBS) supplemented with 0.1% Tween 20 (PBST) for 1 h at room temperature. The sections were then incubated with indicated primary and secondary antibodies with 3 % BSA in PBST, overnight and for 1 h, respectively. Sections were washed with PBST. Rhodamine-labeled PNA (Vector Laboratories, Burlingame, CA) was used to detect the acrosome. Antibodies used are listed in Table S6. Hoechst 33342 (Sigma) was used for counterstaining.

Comet assay

The alkaline comet assay was performed, as described previously (Mori et al.,

2021). In brief, cells were incubated with hydrogen peroxide for 30 min on ice followed by the incubation in GS cell culture medium at 37°C for the indicated time. After centrifugation, cells were embedded in agarose on poly-L-lysine-coated glass slides (Matunami Glass, Osaka, Japan). The cells were lysed by incubating the slides at 4°C in lysis buffer [1% Trinton X-100, 0.5% N-Lauroyl sarcosine sodium salt, 2.5 M NaCl, 100 mM EDTA (pH 8.0), and 10 mM Tris–HCl (pH 10.0)]. After washing, the slides were immersed in pre-chilled electrophoresis buffer (0.3 M NaOH and 1 mM EDTA in water) for 40 min and electrophoresis was performed at 40 V for 50 min at 4°C. Then the samples were washed twice in 400 mM Tris–HCl (pH 7.0) at room temperature and soaked in ethanol for 5 min, followed by overnight drying at 37 ◦C. DNA was detected by ethidium bromide staining for 30 min at room temperature. After rinsing in PBS, slides were observed via confocal microscopy (Fluoview FV3000; Olympus, Tokyo, Japan), and quantified using by Fluoview FV31S-SW software.

Supplemental References

Kanatsu-Shinohara, M., Ogonuki, N., Inoue, K., Miki, H., Ogura, A., Toyokuni, S., and Shinohara, T. (2003). Long-term proliferation in culture and germline transmission of mouse male germline stem cells. Biol. Reprod. *69*, 612-616.

Kanatsu-Shinohara, M., Ogonuki, N., Matoba, S., Morimoto, H., Ogura, A., and Shinohara, T. (2014). Improved serum- and feeder-free culture of mouse germline stem cells. Biol. Reprod. *91*, 88.

Kanatsu-Shinohara M, Shiromoto Y, Ogonuki N, Inoue K, Hattori S, Miura K, Watanabe N, Hasegawa A, Mochida K, Yamamoto T, Miyakawa T, Ogura A, et al. (2023). Intracytoplasmic sperm injection induces transgenerational abnormalities in mice. J. Clin Invest. *133*, e170140.

Martin, M. (2011). Cutadapt removes adapter sequences from high-throughput sequencing reads. EMBnet.J. *17* (10).

Mori, Y., Ogonuki, N., Hasegawa, A., Kanatsu-Shinohara, M., Ogura, A., Wang, Y., McCarrey, J. R., and Shinohara, T. (2021). OGG1 protects mouse spermatogonial stem cells from reactive oxygen species in culture. Biol. Reprod. *104*, 706-716.

Okabe, M., Ikawa, M., Kominami, K., Nakanishi, T., and Nishimune, Y. (1997). 'Green mice' as a source of ubiquitous green cells. FEBS Lett. *407*, 313-319.

Yoshida, K., Hada, M., Hayashi, M., Kizu, A., Kitada, K., Eguchi-Kasai, K., Kokubo, T., Teramura, T., Suzuki, H. H., Watanabe, H., et al. (2024). Transcriptome analysis by RNA sequencing of mouse embryonic stem cells stocked on International Space Station for 1584 days in frozen state after culture on the ground. Int. J. Mol. Sci. 25, 3283.
