# Supplementary material for: Germline transmission of cryopreserved mouse spermatogonial stem cells maintained on the International Space Station
Source: Stem Cell Reports. 2025 Aug 14;20(9):102602. doi: 10.1016/j.stemcr.2025.102602 (PMC12447337; doi:10.1016/j.stemcr.2025.102602)
Supplement: Document S2. Article plus supplemental information [file mmc3.pdf]

Stem Cell Reports
Article
OPEN ACCESS

Germline transmission of cryopreserved mouse spermatogonial stem cells maintained on the International Space Station

Mito Kanatsu-Shinohara,[1,2] Takuya Yamamoto,[3,4,5] Yusuke Shiromoto,[1] Hiroko Morimoto,[1] Tianjiao Liu,[1] Tohru Yamamori,[6] Tomokazu Yamasaki,[7] and Takashi Shinohara[1,8,*]

[1]Department of Molecular Genetics, Graduate School of Medicine, Kyoto University, Kyoto 606-8501, Japan
[2]AMED, 1-7-1 Otemachi, Chiyodaku, Tokyo 100-0004, Japan
[3]Department of Life Science Frontiers, Center for iPS Cell Research and Application, Kyoto University, Kyoto 606-8507, Japan
[4]Institute for the Advanced Study of Human Biology (WPI-ASHIBi), Kyoto University, Yoshida-Konoe, Sakyo, Kyoto 606-8501, Japan
[5]Medical-risk Avoidance based on iPS Cells Team, RIKEN Center for Advanced Intelligence Project (AIP), Kyoto 606-8507, Japan
[6]Space Utilization Promotion Department, Japan Space Forum, Tokyo 101-0062, Japan
[7]Japan Aerospace Exploration Agency, Tsukuba 305-8505, Japan
[8]Lead contact
*Correspondence: tshinoha@virus.kyoto-u.ac.jp
https://doi.org/10.1016/j.stemcr.2025.102602

SUMMARY

Despite growing interest in space exploration, the effects of spaceflight on stem cells remain largely unknown. Damages to germline cells are especially crucial, as spaceflight poses risks to gametogenesis, with abnormalities observed in multiple species. While embryonic stem (ES) cells show genomic instability after space exposure and have not yet produced offspring, this study demonstrates successful offspring production from cryopreserved mouse spermatogonial stem cells (SSCs) stored on the International Space Station for 6 months. Spaceflight did not increase apoptosis or DNA damage in SSCs. After thawing, SSCs proliferated comparably to those cryopreserved on Earth, showing no significant phenotypic or functional differences. Offspring were produced via spermatogonial transplantation followed by natural mating. Since SSCs from many species can be cryopreserved like somatic cells and still produce sperm, they offer a promising resource for germline preservation during space exploration.

INTRODUCTION

It has been over six decades since the first animals were sent into space. Since then, several species, including humans, have experienced long-term spaceflight and safely returned to Earth. However, during spaceflight, stressors, such as radiation, microgravity, hypergravity, and circadian rhythm disruption are inevitable (Mishra and Luderer, 2019). These hazards can lead to varying degrees of organ dysfunction, including stem cells. Common post-flight effects include muscle wasting and decreased bone mineral density, yet the full biological impact of spaceflight remains incompletely understood. Studying stem cells during and after spaceflight is critical, as damage to these cells can result in long-term tissue dysfunction. While most missions have lasted only several months—the longest being 437 days (Kotovskaya et al., 2019)—the prospect of multi-year deep space missions, planetary colonization, and space tourism is becoming realistic. Therefore, it is essential to understand how spaceflight impacts stem cell biology.

Studying germ cells is particularly important since they directly influence the next generation. Given the long lifespan of humans, detailed studies using animals with shorter lifespans are especially valuable in this context. In early studies of rats, no significant changes in the testes were reported (Portugalov et al., 1976; Plakhuta-Plakutina, 1977). However, Fedrova et al. observed a 30%–70% increase in atypical spermatozoa, such as curled tails or the absence of tails, in dogs after 75 days of spaceflight (Fedrova, 1967). Additionally, a 5%–10% decrease in spermatogonia was noted in rat testes during the COSMOS 2044 and COSMOS 1887 missions (Amann et al., 1985; Sapp et al., 1990). More severe damage was observed in mouse testes after 91 days of spaceflight, with experiments revealing significant testicular degeneration and many tubules almost completely devoid of spermatogenic cells, except for a few remaining spermatogonia (Masini et al., 2012). Similar effects were observed in hindlimb suspension models, which simulate spaceflight conditions and lead to abnormal spermatogenesis (Hargens et al., 1984; Tou et al., 2002). Immobilization of primates also resulted in the arrest of spermatogenesis (Zemjanis et al., 1970). Thus, abnormal spermatogenesis in space likely affects many animal species, including humans.

Due to these dramatic environmental changes, analyzing physiological functions in animals during spaceflight is complicated by both systemic and local factors. For example, in some studies, over half of the experimental mice died due to spaceflight-related stresses (Masini et al., 2012). Some systemic effects are mediated by disruptions in the hypothalamic-pituitary-testis axis. Serum, urine, and salivary testosterone levels significantly decreased during flight compared to pre-flight levels (Mishra and Luderer, 2019; Ahrari et al., 2022). Animals also exhibited

© 2025 The Author(s).
Published by Elsevier Inc. on behalf of International Society for Stem Cell Research.
This is an open access article under the CC BY license (http://creativecommons.org/licenses/by/4.0/).

increased serum luteinizing hormone levels, consistent with decreased negative feedback from the testes. Interestingly, testosterone levels in serum or intratesticular tissue did not seem to be affected by irradiation (Cunningham and Huckins, 1978), suggesting that spaceflight-induced stress is more severe than radiation alone. In addition to hormonal changes, local testicular cell-cell interactions also affect germ cell survival. Gene expression in Sertoli cells, which are in direct contact with germ cells, is influenced by microgravity (Cirelli et al., 2017). While the risks of spaceflight are well known, most studies have focused on damage to somatic cells, and much remains to be learned about the mechanisms behind abnormal spermatogenesis during spaceflight.

One potential approach to addressing these issues is to examine the function of pure germ cells stored in space. Sperm cells are likely the most resistant cell type in the body, capable of surviving and maintaining fertility even after exposure to 25 Gy (Wakayama et al., 2021). This is in contrast to most somatic cells, which undergo apoptosis when exposed to 1–2 Gy. Leveraging this resilience, Wakayama et al. reported in 2017 that freeze-dried mouse spermatozoa exposed to space ionizing radiation for 288 days on the International Space Station (ISS) showed no significant changes in fertilization or birth rates (Wakayama et al., 2017). Although a slight increase in sperm DNA damage was observed, fertilization was still successful via intracytoplasmic sperm injection (ICSI) into oocytes. These findings are promising and suggest freeze-drying as a potential method for germline conservation. Offspring were successfully produced from freeze-dried sperm stored on the ISS for up to 5 years and 10 months. However, ICSI affects genomic imprinting and may lead to behavioral and morphological abnormalities (Fernández-González et al., 2008; Esteves et al., 2018; Lewon et al., 2020; Kanatsu-Shinohara et al., 2023), and extended analysis is required for understanding the impact of using freeze-drying sperm.

Previous studies report detrimental effects of spaceflight on stem cells. For example, while embryonic stem (ES) cells may serve as an alternative resource for germline preservation, several groups report the upregulation of apoptosis- and senescence-associated genes, and no offspring are born from ES cells stored in space (An et al., 2019; Yoshida et al., 2024; Mao et al., 2024). Specifically, the downregulation of DNA repair genes has raised concerns about potential germline damages. It is possible that embryonic cells are particularly sensitive to spaceflight because preimplantation embryos exhibited significantly poor *in vitro* development during spaceflight, likely due to irradiation (Lei et al., 2020). Another study also reported ectopic expression of an inner cell mass marker in the blastocyst cavity (Wakayama et al., 2023). As a result, currently artificial reproductive technologies may have limited applications for germline preservation in space, raising concerns about reproductive protection during human spaceflight.

Spermatogenesis begins with spermatogonial stem cells (SSCs), which continuously self-renew and produce numerous sperm (Meistrich and van Beek, 1993; de Rooij and Russell, 2000). As they differentiate, SSCs give rise to differentiating spermatogonia, which are particularly sensitive to radiation (De Felice et al., 2019). This high sensitivity may explain the significant loss of spermatogonia observed in mouse testes during spaceflight. One unique characteristic of SSCs is their ability to increase in number through stimulated self-renewal. The addition of glial cell line-derived neurotrophic factor (GDNF) and fibroblast growth factor (FGF)2, both key factors in SSC self-renewal, allows for the *in vitro* expansion of cultured SSCs, designated as germline stem (GS) cells, for over 2 years (Kanatsu-Shinohara et al., 2003a; Kanatsu-Shinohara and Shinohara, 2013). This provides a new opportunity, as SSCs, unlike sperm, undergo meiosis, providing a unique resource for generating genetic diversity. Another advantage of SSCs is their straightforward freezing protocol (Avarbock et al., 1996). In contrast, sperm cryopreservation requires species-specific protocols due to the unique head morphology of sperm (Holt, 2000), making it difficult to establish a universal method. However, SSCs from various species, including humans, can be frozen similarly to somatic cells. Offspring have been produced by transplantation of cryopreserved SSCs by natural mating (Kanatsu-Shinohara et al., 2003b).

In this study, we utilized SSCs for long-term cryopreservation during spaceflight. GS cells were frozen for 6 months aboard the ISS. Although live animals have been maintained on the ISS for up to 3 months (Cancedda et al., 2012), the analysis of germ cells in these animals remains limited due to the challenges of long-term maintenance of live animals in space. Since frozen cells lack DNA repair mechanisms, they offer a sensitive experimental system to study potential damage in SSCs. Upon return to Earth, the frozen SSCs were thawed, and their DNA damage and gene expression profiles were analyzed. The impact on SSC activity was further evaluated through spermatogonial transplantation into infertile mouse testes.

RESULTS

Survival of SSCs after cryopreservation and irradiation on Earth

Since cryopreservation inhibits the DNA repair mechanisms, we first assessed the effects of cryopreservation and irradiation on SSCs on Earth. Four types of GS cells were used: fresh non-irradiated, fresh irradiated, frozen non-irradiated, and frozen irradiated GS cells. Irradiation

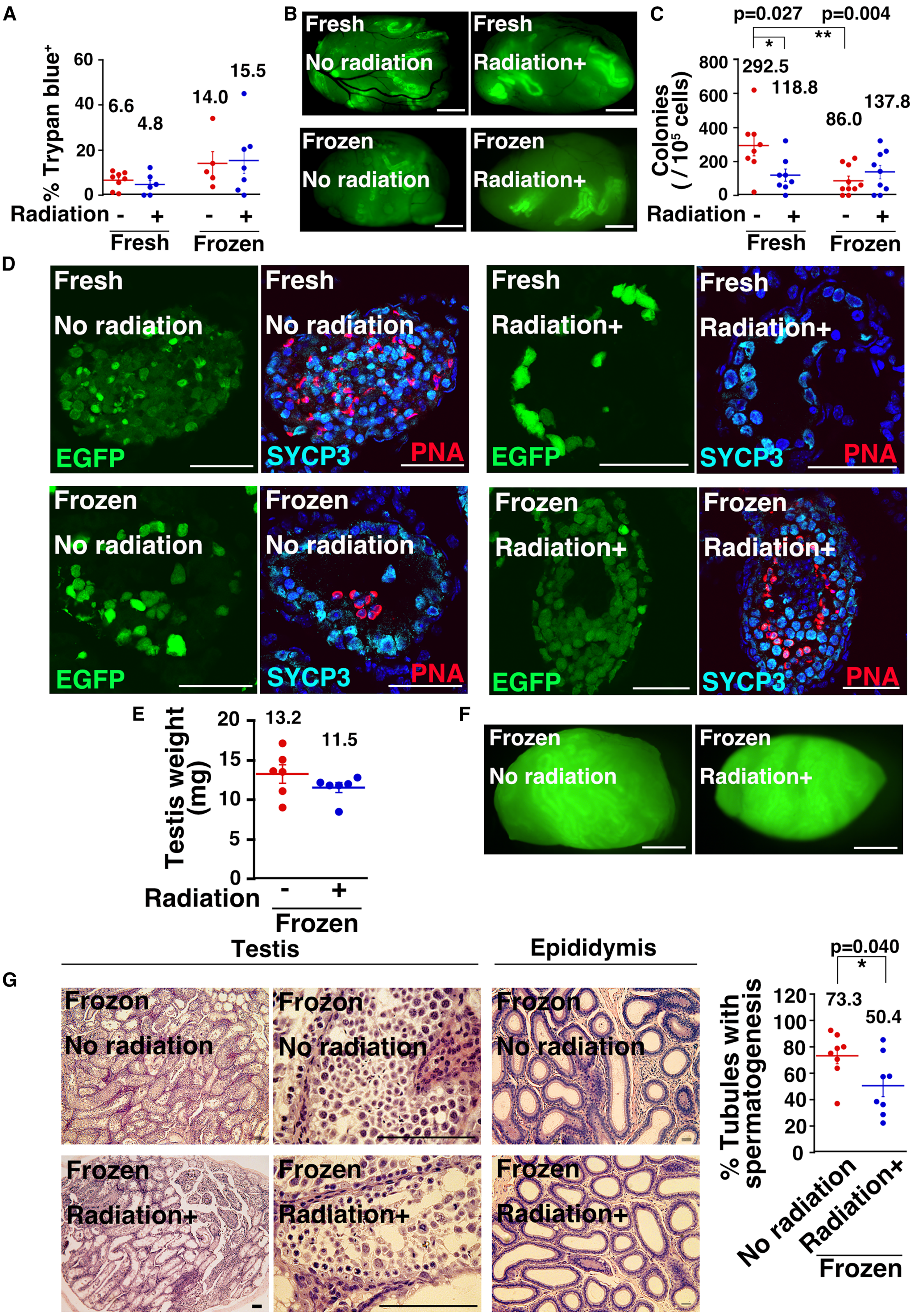

(legend on next page)

involved exposing dissociated GS cells to 1.5 Gy, the median lethal dose 50 for GS cells (Ishii et al., 2014a; 2014b). Cryopreservation was performed in a DMSO-based solution. To evaluate cell viability, we used trypan blue staining, which showed that both frozen groups had a higher proportion of dead cells compared to fresh groups (Figure 1A), but no significant differences were found among the four samples.

Two experiments were conducted using spermatogonial transplantation to further evaluate SSCs (Brinster and Zimmermann, 1994). In the first experiment, SSC frequency was determined by counting colonies in busulfan-treated mice, which have empty tubules. Two months after transplantation into busulfan-treated mice, the mice were sacrificed, and the number of colonies was counted (Figure 1B). Colony numbers were 292.5, 118.8, 86.0, and 137.8 per 10^5 transplanted cells, for fresh, fresh irradiated, frozen, and frozen irradiated cells, respectively (Figure 1C). While survival rates were comparable, fresh cell transplantation produced significantly more colonies, and irradiation reduced the number of colonies to 40.6% in fresh GS cells, while frozen cells showed a 60.2% increase in colony formation post-irradiation. However, there was no statistical significance. Immunostaining of recipient testes showed that irradiated fresh cells produced very few tubules with peanut agglutinin $(PNA)^+$ haploid cells, though these tubules contained $SYCP3^+$ meiotic spermatocytes (Figure 1D).

In the second set of experiments, we aimed to confirm the extent of complete differentiation, as this information is critical for producing offspring through natural mating. We used congenitally infertile WBB6F1-W/W^v (designated W) mice and compared irradiated frozen GS cells with non-irradiated frozen cells. Unlike busulfan-treated mice, W mice lack endogenous spermatogenesis due to mutations in the *Kit* tyrosine kinase receptor (Fleischman, 1993). By transplanting a large number of GS cells, we were able to evaluate the donor cells' complete differentiation capacity. Three months post-transplantation, there was no difference in testis weight between the two groups (Figure 1E). Due to the higher number of transplanted cells (4×10^5 cells per testis), donor cell differentiation enhanced in the recipient testes, as indicated by long green segments of seminiferous tubules under UV light (Figure 1F). Histological analysis showed complete spermatogenesis in both groups (Figure 1G). However, the number of tubules undergoing spermatogenesis was lower following transplantation of irradiated frozen GS cells compared to non-irradiated GS cells. Given that the number of germ cell colonies did not differ significantly (Figure 1C), this result suggests that regeneration is delayed by irradiation. Nevertheless, the presence of spermatozoa in the epididymides of all recipients indicates that they may still be capable of achieving fertility through natural mating.

Evaluation of total space radiation

For storage on the ISS, GS cells were cryopreserved and stored in the Minus Eighty-Degree Laboratory Freezer (MELFI) freezer for 6 months. A control set of GS cells was also cryopreserved and stored in the MELFI freezer on Earth. The samples were launched to the ISS on July 14, 2022, and returned to Earth on January 11, 2023, remaining in space for a total of 181 days. The cells were kept at $-95°C$ during the spaceflight and at $-80°C$ during transport on Earth. Upon arrival in Kyoto, the cells were stored in liquid nitrogen for subsequent analysis.

To evaluate the radiation dose, we analyzed the bio passive dosimeter for life science experiments in space (PADLES) (Figure 2A), which contains CR-39 plastic nuclear track detector (PNTD) for measuring radiation dose and characteristics (Nagamatsu et al., 2013). PADLES was placed near the frozen samples to accurately measure the cosmic radiation dose. Analysis of the PADLES surface revealed etch marks from nuclear tracks created by atomic nuclei during spaceflight. The linear energy transfer (LET) distribution was determined by subtracting the background LET distribution measured with PNTDs from the ground control package at the Tsukuba Space Center (TKSC). According to PADLES, the absorbed radiation dose rate was 0.31 ± 0.01 mGy per day, corresponding to

Figure 1. Impact of radiation on frozen GS cells

(A) Cell survival rate after freezing and radiation ($n = 8$, 6, 5, and 7 experiments for fresh, fresh radiation, frozen, and frozen radiation, respectively).

(B) Macroscopic appearance of busulfan-treated recipient testis transplanted with GS cells.

(C) Colony counts ($n = 8$, 8, 10, and 9 testes for fresh, fresh radiation, frozen, and frozen radiation, respectively).

(D) Immunostaining of recipient testis for SYCP3 (spermatocyte marker) and PNA (haploid cell marker).

(E) Testis weight ($n = 6$ testes).

(F) Macroscopic appearance of W recipient testis transplanted with GS cells.

(G) Histological appearance of recipient testis and epididymis. 824 and 874 tubules were counted in 8 testes for no radiation and radiation, respectively.

Stain, hematoxylin and eosin (G) and Hoechst 33342 (D). Bar = 1 mm (B and F), 50 μm (D), and 100 μm (G). Each bar represents the mean ± SEM, and the numerical mean value is indicated above each bar. $*p < 0.05$; $**p < 0.01$.

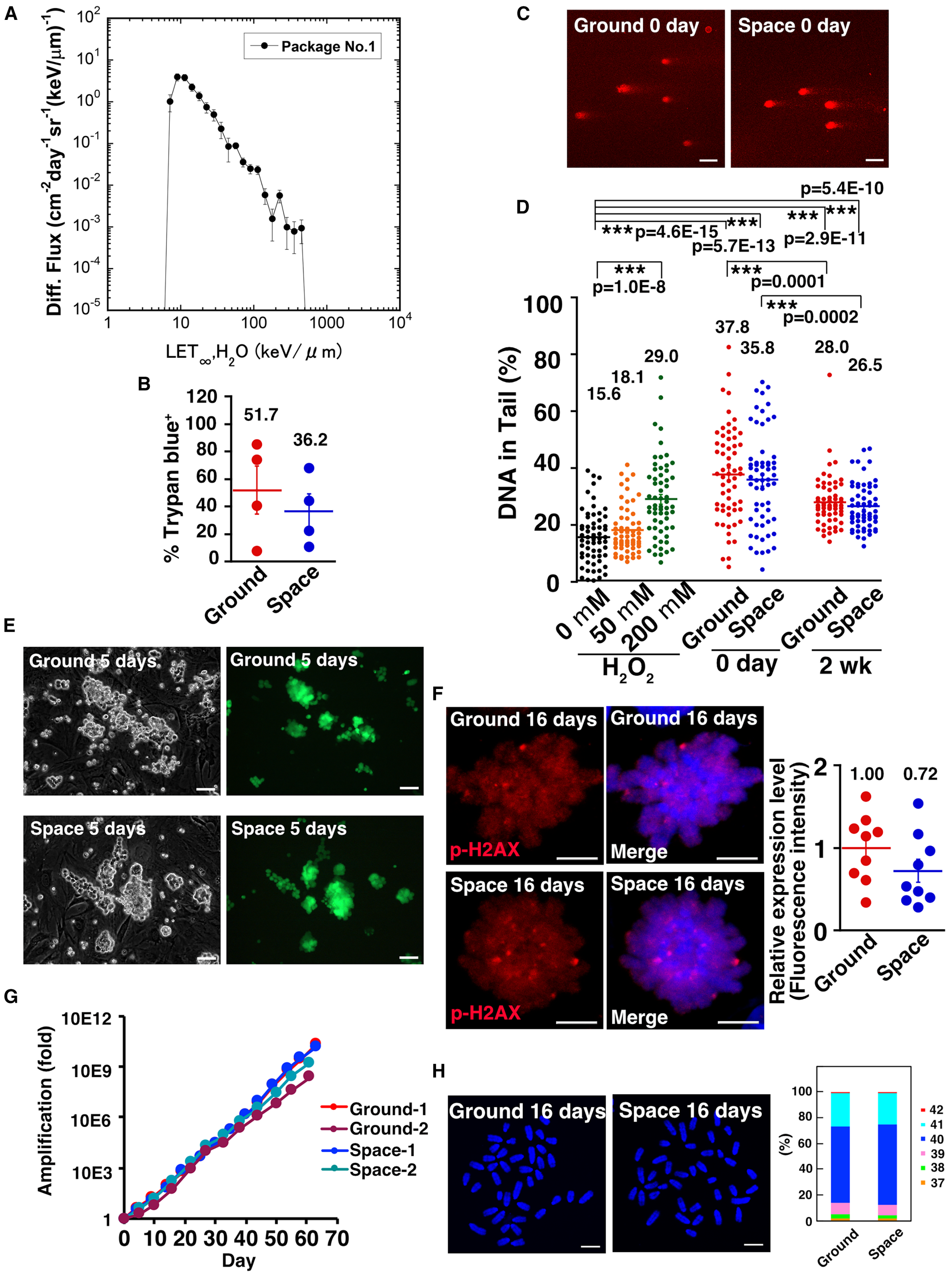

(legend on next page)

a dose-equivalent rate of 0.59 ± 0.03 mSv per day. The total absorbed dose was 106.5 ± 5.2 mSv.

We thawed the cells and assessed survival using trypan blue staining, with approximately 50%–60% of the cells surviving post-thaw (Figure 2B). No significant differences were observed between GS cells stored in space (Space GS cells) and those on Earth (Ground GS cells) (Figure 2B). We also conducted an alkali comet assay to detect both single-strand and double-strand DNA breaks (Figure 2C). As a positive control, we treated GS cells with hydrogen peroxide at two different doses (50 and 200 μM) for 30 min.

Unexpectedly, analysis of Space GS cells did not show an increase in DNA damage compared to Ground GS cells. However, both Space and Ground GS cells exhibited significantly more DNA damage than fresh GS cells cultured without hydrogen peroxide. Because Space GS cells have undergone exposure to damages by spaceflight, this result suggested that the damage by spaceflight was limited compared to freeze-thawing process. When reanalyzed 2 weeks after thawing and two rounds of passages, both types of cells showed significantly reduced DNA damage compared to freshly thawed cells. Nevertheless, the damage was not completely repaired, as more DNA damage was present than in fresh GS cells. Both types of cells exhibited noticeable DNA damage upon thawing, but no difference was found in the intensity ratio of head over total DNA between the two samples (Figures 2C and 2D).

When Space GS cells were cultured *in vitro*, their colony morphology was indistinguishable from Ground GS cells (Figure 2E), showing grape-like clusters of spermatogonia. Immunostaining of cells cultured for 16 days after thawing and two passages revealed that the expression levels of phosphorylated-H2AX, a marker of DNA damage, were comparable between Ground and Space GS cells (Figure 2F). These cells proliferated normally for at least 100 days. The proliferation rate was comparable between Space and Ground GS cells, with doubling times of 2.0 and 1.8 days, respectively (Figure 2G). Since an abnormal karyotype can impair spermatogenesis and cause infertility, we also evaluated the karyotype of Space GS cells 16 days after thawing with two rounds of passages, revealing that 63% of the cells had 40 chromosomes (Figure 2H). The difference was not statistically significant.

Gene expression and DNA methylation in Space GS cells

To assess the phenotype of Space GS cells, we first performed flow cytometry with antibodies against spermatogonia surface markers. A comparison between Space and Ground GS cells showed no significant differences in the mean fluorescence intensity for the tested antigens (Figures 3A and 3B). Considering previous reports on ES cells that indicated downregulation of DNA repair genes and upregulation of senescence- and apoptosis-associated genes (An et al., 2019; Mao et al., 2024), we conducted RNA sequencing (RNA-seq) to identify genes specifically induced by spaceflight. Using three lines of Space and Ground GS cells, we performed hierarchical clustering analysis, but no significant differences in gene expression profiles were observed (Figure 3C; Table S1). Real-time PCR analyses showed no significant changes in the expression levels of *Trp53*, *Rad51*, and *Pten* (Figure 3D).

Additionally, we conducted reduced representation bisulfite sequencing (RRBS) to examine potential changes in DNA methylation patterns (Figures 3E and S1). Among 681,804 covered CpG sites covered in gene promoters, our analysis identified 770 hypermethylated sites (0.113% of commonly covered sites) and 563 hypomethylated sites (0.083% of commonly covered sites) in Space GS and Ground GS cells, respectively (>20% change, R^2 = 0.9912; Table S2). There were no significant differences in the overall DNA methylation of promoter CpG sites between Space GS cells and Ground GS cells (Figure S1). Only four genes were differentially methylated between

Figure 2. Phenotype of Space GS cells

(A) Differential particle flux function of LET, H_2O measured with CR-39 PNTDs in flight package no. 1 measured by PADLES. This LET distribution was obtained by subtracting the background LET distribution measure with the CR-39 PNTDs in TKSC ground control package no. 2.

(B) Viability of GS cells by trypan blue staining, immediately after thawing (n = 4 experiments).

(C and D) Comet assay (C) and quantification (D) on the day of thawing. The head (round shape) corresponds to undamaged DNA, while the tail (smear) corresponds to damaged DNA. Sixty cells were picked up, and the intensity ratio of head over total DNA was quantified.

(E) Appearance of Space GS cells *in vitro* 5 days after thawing.

(F) Immunostaining of Space GS cells 16 days after thawing with two rounds of passages. Nine cells at metaphase stage were analyzed for p-H2AX expression level.

(G) Proliferation of Space GS cells.

(H) Karyotype of Space GS cells 16 days after thawing with two rounds of passages. One hundred cells were analyzed for counting chromosome number.

Bar = 50 μm (C and E) and 5 μm (F and H). Each bar represents the mean ± SEM, and the numerical mean value is indicated above each bar. $^{***}p < 0.001$.

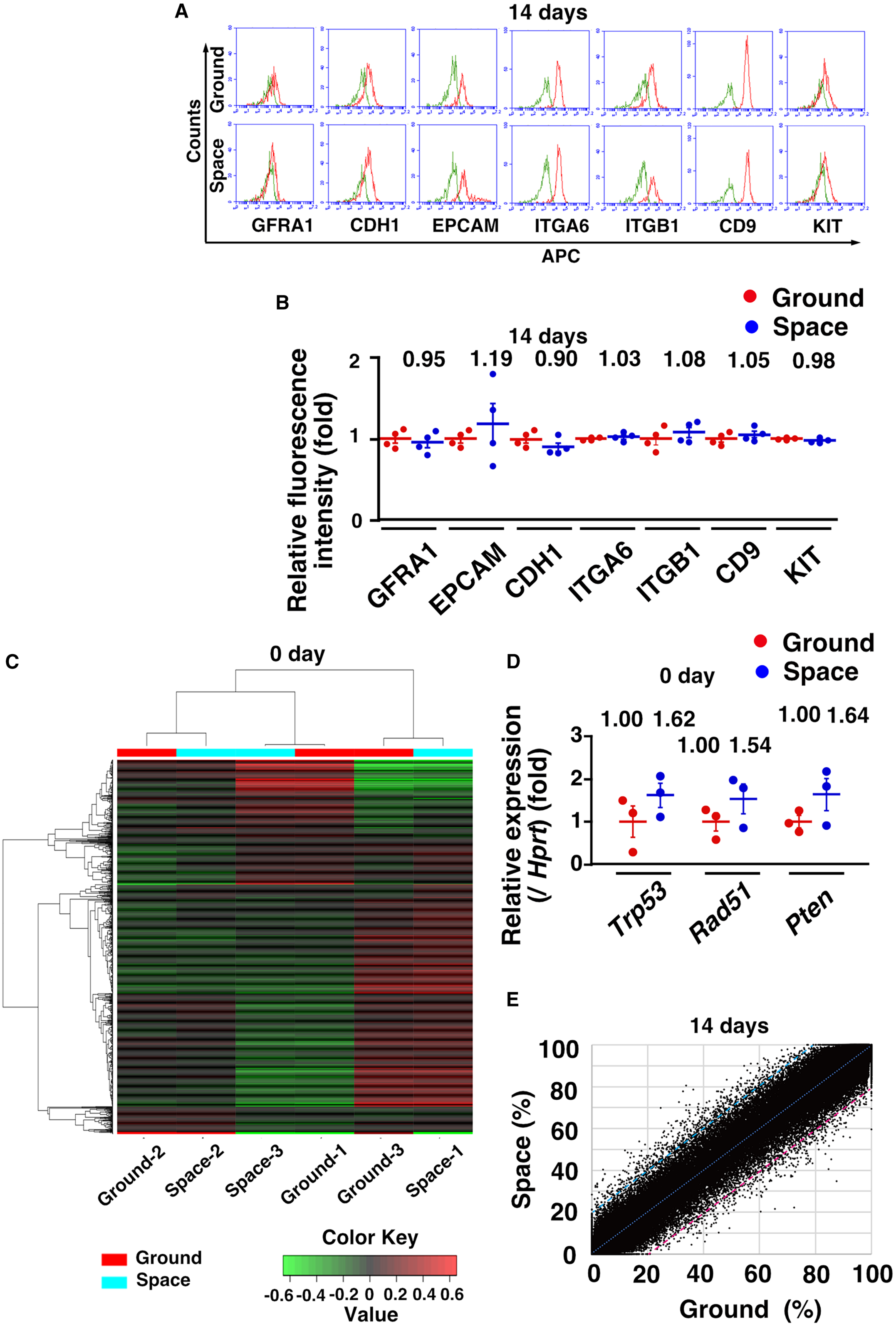

(legend on next page)

the two groups: *Gm7097*, *Bsnd*, and *Fgf21* were hypermethylated in Space GS cells, while *Gm15562* was hypomethylated in ground GS cells. *Fgf21* was downregulated in Space GS cells, while *Gm15562* was expressed only in Ground GS cells according to RNA-seq data (Table S3). The two other genes were not expressed in either type of GS cells. No significant differences in DNA methylation patterns were observed in imprinted genes. These findings suggest that the phenotype of GS cells did not change significantly after spaceflight.

Functional analysis of space GS cells by spermatogonial transplantation

Since only a portion of GS cell contain SSCs (Kanatsu-Shinohara and Shinohara, 2013), we performed spermatogonial transplantation experiments during 3 months of culture. Both types of cells proliferated at a comparable rate, with no significant changes in growth characteristics. The cells were transplanted into the seminiferous tubules of busulfan-treated mice at 26, 63, and 97 days after thawing. When the number of colonies was counted 2 months post-transplantation, we observed significant increases in the number of germ cell colonies at 63 and 97 days compared to 26 days (Figures 4A and 4B). Although no significant difference between Ground GS cells and Space GS cells was noted at 26 days, both exhibited poor colonization levels compared to longer-term cultures. However, these values were within the previously reported results, which ranged from 2.0 to 800.0 per 10^5 cells (Kanatsu-Shinohara et al., 2005; Miyazaki et al., 2023). SSCs increased logarithmically at comparable rates (Figure 4C; Table S4). These results suggest that spaceflight did not affect the balance between SSCs and progenitors *in vitro*.

Based on these observations, we next examined the feasibility of producing offspring from Space GS cells. Immature W pups were used as recipients, as donor cells colonized these recipients 5 to 10 times more efficiently than adult W mice (Shinohara et al., 2001). The absence of a blood-testis barrier in immature testes is believed to enhance SSC homing to germline niches (Kanatsu-Shinohara et al., 2020). After transplantation, the pups were returned to their mothers for sexual maturation. At about 1 month of age, the recipient mice were housed with wild-type C57BL/6 (B6) females. One of the offspring became fertile as early as 89 days after transplantation. Although no donor fluorescence was observed in the first litter, $EGFP^+$ offspring were found in the second litter, confirming the donor cell origin of the offspring (Figure 4D; Space pups). Overall, 3 of 8 mice became fertile from Space GS cells. Offspring were also obtained from Ground GS cells (Ground pups) as early as 88 days after transplantation, with 4 of 8 recipients becoming fertile. The number of days to the first offspring, the total number of litters, and the litter size did not show significant differences during ∼5 months of natural mating (Figures 4E–4G).

Six months after transplantation, the mice were sacrificed, and their testes were analyzed (Figure 4H). No significant differences were observed in testis size between the two groups (Figure 4I). Histological examination of the testes revealed normal spermatogenesis in both groups (Figure 4J). Quantification of seminiferous tubules with spermatogenesis showed no significant differences between Space and Ground GS cells (Figure 4K). While sperm were present in the epididymides of fertile recipients, they were similarly found in 50% of the epididymides of infertile recipients (Figure 4J). Immunostaining of recipient testes confirmed the presence of $SYCP3^+$ spermatocytes and PNA^+ haploid cells, with no morphological abnormalities (Figure 4L). These results suggest that transplantation of a larger number of cells might have allowed offspring production.

Analysis of the offspring

Given that ICSI can cause abnormalities in genomic imprinting (de Waal et al., 2012), we used combined bisulfite restriction analysis (COBRA) to examine the genomic imprinting of the offspring using tail DNA. Differentially methylated regions (DMRs) of the *H19* and *Igf2r* genes were analyzed. All offspring exhibited normal somatic DNA methylation patterns, with no apparent differences

Figure 3. Analysis of Space GS cells

(A) Flow cytometric analysis using antibodies against spermatogonia markers. Green lines indicate controls. The cells were analyzed 14 days after thawing with two rounds of passages.

(B) Mean fluorescence intensity (n = 4 experiments).

(C) Heatmap of RNA-seq. The cells were analyzed on the day after thawing without passages.

(D) Real-time PCR analysis immediately after thawing (n = 3).

(E) A scatterplot showing correlation of the DNA methylation data at individual CpG sites in gene promoters (n = 3 experiments). The numbers of identified hypermethylated sites and hypomethylated sites in Space and Ground GS cells are shown in red and blue, respectively, along with the percentages of commonly covered sites. Red or blue lines indicate 20% increased methylation levels or 20% decreased methylation levels in Space GS cells, respectively. The dashed line indicates the linear regression line. The cells were analyzed 14 days after thawing with two rounds of passages. Each bar represents the mean ± SEM, and the numerical mean value is indicated above each bar. See also Figure S1.

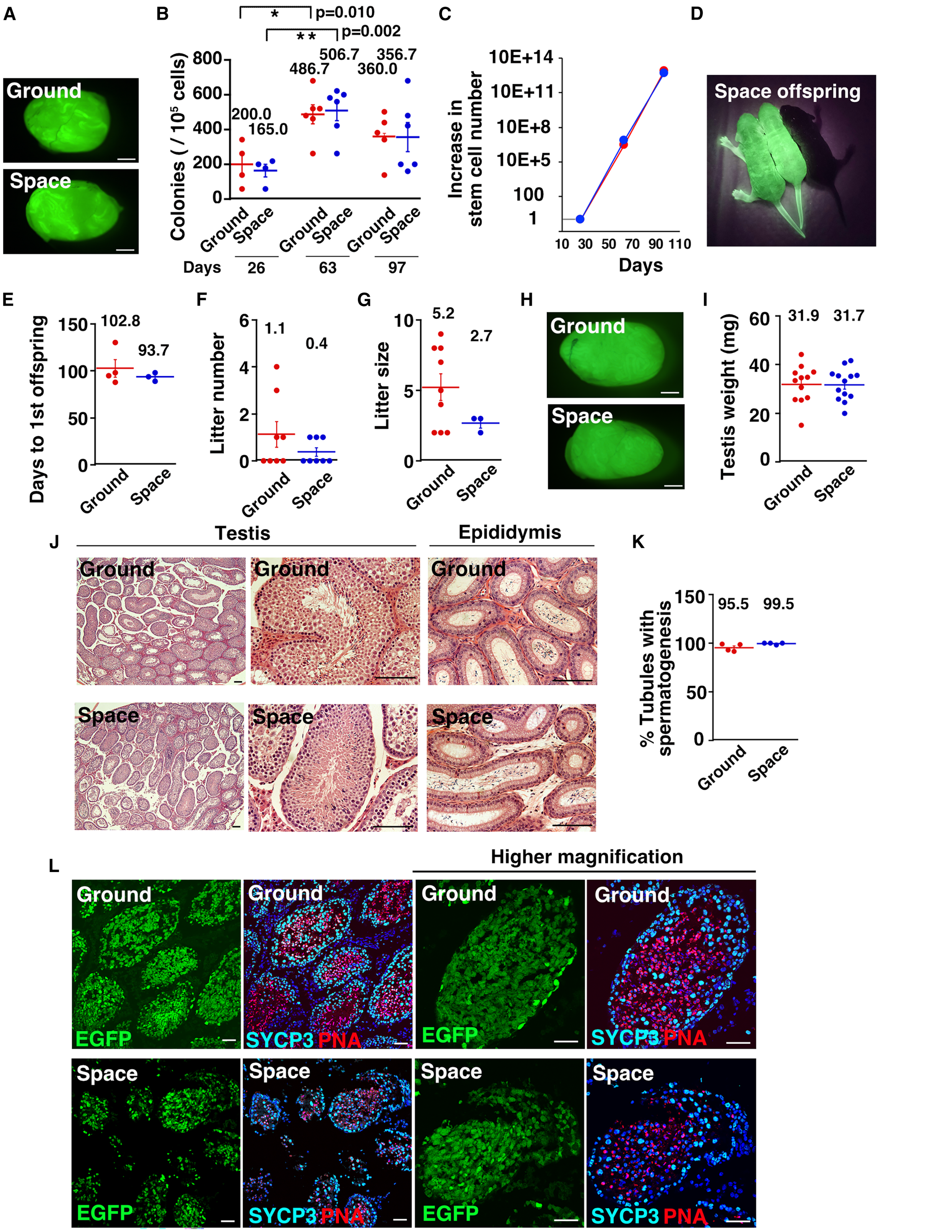

(legend on next page)

between the two types of donor cells (Figure S2A). Bisulfite sequencing further confirmed no abnormalities in the DMRs of these genes (Figure S2B). However, because abnormalities may not be evident by DNA methylation, RNA-seq analysis of liver tissue was performed to verify normal gene expression patterns in Space pups, Ground pups, and wild-type mice born through natural mating. Three F1 males and females from each group were analyzed and compared with three wild-type mice of the same genetic background (Table S5). No significant differences in gene expression were observed among the groups (Figure S2C). These findings suggest that Space GS cells retain fertility and produce normal offspring.

DISCUSSION

This study has two primary objectives. The first is to evaluate the utility of SSCs for germline preservation in space. Although freeze-dried sperm has been successfully stored and used during spaceflight (Wakayama et al., 2017, 2021), ICSI and freeze-drying procedure may pose long-term potential health risks to offspring. The second objective is to assess potential damage to SSCs caused by cosmic radiation. Spermatogenesis is significantly impaired post-spaceflight, including a reduction in the number of spermatogonia (Mishra and Luderer, 2019), suggesting that SSCs could be particularly sensitive to cosmic radiation. Unlike differentiated germ cells, which differentiate and disappear, any damage to SSCs could persist long-term and accumulate in the germline. Thus, preventing genomic damages in SSCs is crucial for safe spaceflight. In this context, evaluating the fertility of frozen SSCs not only serves as a new biological resource for germline preservation but also helps assess spaceflight-related risks.

Our initial Earth-based experiments showed that frozen GS cells are relatively resistant to irradiation. While DNA repair mechanisms do not function in frozen cells, it has been previously suggested that frozen cells are better protected from irradiation than fresh cells (Aschwood-Smith and Friedmann, 1979; Cugia et al., 2011). These studies reveled a 3.5- to 20-fold higher survival rate for frozen cells stored in liquid nitrogen compared to fresh cells. This observation aligns with finding of fewer gammaH2AX foci (a marker of double-strand DNA breaks) in frozen irradiated cells compared to fresh irradiated cells. Although the exact reason for this improved survival is unclear, it has been proposed that the production and diffusion of reactive oxygen species (ROS), which can damage DNA and cell structures, are reduced in the frozen state (Cugia et al., 2011). In our study, however, we did not observe improved survival rates in irradiated frozen GS cells. This may be due to the storage conditions: ideally, cells should be stored at $-196^{\circ}C$ in liquid nitrogen, whereas we used a deep freezer due to the unavailability of liquid nitrogen on the ISS. Nevertheless, the relatively high resistance of frozen GS cells to irradiation formed the basis for our space-based experiments.

Our analysis of Space GS cells revealed no significant increase in damage using the comet assay. Given the notable damage observed in ES cells and early embryos exposed to spaceflight (An et al., 2019; Lei et al., 2020; Wakayama et al., 2023), we expected GS cells would show heightened apoptosis, as they are more vulnerable to DNA double-strand breaks compared to ES cells (Ishii et al., 2014a, 2014b). This is not surprising because ES cells are notorious for their high sensitivity to radiation (Aladjem et al., 1998; Hong and Stambrook, 2004). Contrary to these expectation, the comet assay showed that cryopreservation caused more damage than spaceflight in GS cells. Hydrogen

Figure 4. Functional analysis of Space GS cells

(A) Macroscopic appearance of adult W testis transplanted with Space GS cells.

(B) Colony count (n = 4, 6, and 5 [ground] and 4, 6, and 6 [space] testes for 26, 63, and 97 days, respectively).

(C) Increase in SSC number from the first transplantation. To evaluate the total stem cell number during culture, we transplanted a portion of cells at the time of passages into infertile mice. The total number of SSCs was estimated by multiplying the total cell counts by the stem cell concentration, as determined by transplantation assay.

(D) Offspring born from W recipient transplanted with Space GS cells, showing donor cell-derived green fluorescence.

(E) Days to first offspring (n = 4 mice for Ground; n = 3 mice for Space).

(F) Litter number (n = 8 experiments).

(G) Litter size (n = 9 experiments for Ground; n = 3 experiments for Space).

(H) Macroscopic appearance of pup W testis transplanted with Space GS cells.

(I) Testis weight (n = 12 testes for Ground, n = 13 testes for Space).

(J) Histological appearance of testis and epididymis. Epididymides were from infertile recipients.

(K) Frequencies of tubules with spermatogenesis. 402 and 352 tubules in 4 testes were counted for Ground and Space GS cell recipients, respectively.

(L) Immunostaining of W mouse testis for SYCP3 (spermatocyte marker) and PNA (haploid cell marker).

Bar = 1 mm (A and H), 100 μm (J), and 50 μm (L). Each bar represents the mean ± SEM, and the numerical mean value is indicated above each bar. *$p < 0.05$; **$p < 0.01$. See also Figure S2.

peroxide concentration used (50 μM) was sufficient to kill both mouse embryonic fibroblasts and ES cells in a week (Mori et al., 2021), yet only minimal differences were observed between Space GS cells and Ground GS cells, suggesting that cells in the cryopreserved state have some specific protective mechanism against radiation. Although radiosensitivity in ES cells can be improved by overexpression of *Chk2* gene by restoring a G1 arrest (Hong and Stambrook, 2004), this may not apply to GS cells because GS cells have a long G1 phase (Ishii et al., 2014a, 2014b). Instead, since GS cells possess strong base excision repair (BER) activity (Mori et al., 2021), this may have helped protect them from spaceflight-induced damage, differentiating them from ES cells, which have weaker BER activity (Mori et al., 2021).

Further analysis revealed that GS cells proliferated normally *in vitro*. After spermatogonial transplantation, similar numbers of germ cell colonies were observed between Space GS cells and Ground GS cells. Though smaller numbers of colonies were formed 26 days post-culture initiation, this could reflect freeze-thaw damage as indicated by the comet assay (Figure 2C). Despite this initial difference, the number of colonies remained constant, and their morphology showed no substantial differences between the two groups. Over 3 months, there were no significant changes in SSC numbers, indicating that cosmic rays did not disrupt the balance between self-renewal and differentiating in SSCs. Therefore, spaceflight did not impair the SSCs' ability to recolonize the seminiferous tubules. These results contrast with studies on neural stem cells (NSCs), which showed an 8-fold increase in cell-cycle activity after returning to Earth following space exposure, which normalized after 30 h of re-exposure to 1*g* condition (Shaka et al., 2022). These NSCs demonstrated "memory" effects post-spaceflight, experiencing abnormal cell division patterns, including incomplete or muti-daughter cell divisions. Additionally, spaceflight downregulated the GDNF signaling pathway in ES cells. Because GDNF is an essential self-renewal factor for SSCs, this raised concerns that GS cells may have difficulty responding to self-renewal signals upon thawing (An et al., 2019). However, the normal proliferation of GS cells after spaceflight suggests that self-renewal regulation resumed without issues.

The most notable result of this study was the successful production of offspring through natural mating. These offspring were born within 3–4 months after spermatogonial transplantation, consistent with the expected timeline for fertility restoration following such transplants (Shinohara et al., 2001; Kanatsu-Shinohara et al., 2016). Importantly, the offspring from Space GS cells showed no noticeable congenital defects, normal genomic imprinting patterns, and no significant differences in gene expression. Further analyses, such as lifespan assessments and fertility testing, are required to rule out long-term health issues. In particular, although offspring produced by ICSI did not show any abnormal DNA methylation patterns, additional analyses are required to confirm the long-term health of the offspring as well as their descendants. Nevertheless, the birth of normal-appearing offspring underscores the potential utility of SSCs for germline conservation.

At least two questions arise from the present study. First, it will be crucial to determine how long SSCs can be kept frozen on the ISS. Since the cells are in a frozen state, potential damage from cosmic ray will accumulate without DNA repair mechanisms, potentially compromising SSC function. Although the differences were not statistically significant, analysis of *Trp53*, *Rad51*, and *Pten* mRNA levels revealed increased expression in Space GS cells (Figure 3D). Extended storage on the ISS may lead to greater damage and compromise their reproductive potential. Such damage to SSCs could pose a critical concern for human spaceflight, as human spermatogenesis is generally more sensitive to irradiation than that of mice (Clifton and Brenner, 1983). Second, assessing the impact of genomic damages on the offspring and subsequent generations is essential. Although apparently healthy offspring were born, it is unclear whether spaceflight-induced DNA damages were fully repaired. Previous studies have shown elevated hepatic expression of DNA replication-related genes in offspring produced via *in vitro* fertilization using sperm from space-exposed males (Yoshida et al., 2021). Additionally, mice produced via ICSI using freeze-dried sperm after spaceflight had a shorter lifespan than typical B6 mice (604 days vs. 901 days) (Wakayama et al., 2021). Long-term observation is crucial to understand the effects of prolonged cosmic ray exposure.

In conclusion, cosmic influences on stem cells are a critical issue for long-term human space habituation. The successful production of offspring from cryopreserved SSCs demonstrates the utility of SSCs for germline preservation in space. By using spermatogonial transplantation, it is possible to distinguish the effects of spaceflight on germ cells and somatic cells. However, while healthy offspring were obtained, much remains to be explored regarding the risks of spaceflight on SSCs. Given the long reproductive lifespan of humans, animal models with shorter lifespans are essential for such research. As the space industry continues to expand, these experiments are necessary prerequisites for future long-term human spaceflights. The next logical step would be to culture GS cells on the ISS and monitor the impact of cosmic radiation on live germ cells, which may provide further insights into DNA repair mechanisms and enhance the technique's applicability for space missions. Thus, our successful production of

offspring from cryopreserved SSCs offers a solid foundation for future space-based applications.

METHODS

Animals and transplantation procedure

For colony counting, cells were microinjected into the seminiferous tubules of mature B6 × DBA/2 F1 (BDF1) mice (Japan SLC, Shizuoka, Japan). These mice received intraperitoneal injection of busulfan (44 mg/kg; Sigma, St. Louis, MO) to remove endogenous spermatogenesis at 4 weeks of age and were used at least 1 month after busulfan treatment. For assessment of spermatogenesis differentiation levels, 4- to 8-week-old W mice were used. For fertility experiments, we used 5- to 10-days-old W pups (Japan SLC), as described previously (Shinohara et al., 2001). All injection was performed via efferent duct, and 75%–85% of the tubules were filled with the cell suspension, as described previously (Ogawa et al., 1997). Approximately 10 μL was microinjected into BDF1 testis, whereas 2 μL was injected into W pups because they were smaller. The Institutional Animal Care and Use Committee of Kyoto University approved all animal experimentation protocols.

COBRA

Genomic DNA was treated with sodium bisulfite, which deaminates unmethylated cytosines to uracils but does not affect 5-methylated cytosines. Bisulfite-treated DNA was used as a template for amplifying DMRs using specific primers. The amplified DNA was cut with methylation-sensitive restriction enzymes, as described previously (Kanatsu-Shinohara et al., 2004). The PCR primers used in the experiments are shown in Table S7.

Bisulfite sequencing

DNA methylation analysis was performed as described previously (Kanatsu-Shinohara et al., 2023). Bisulfite-treated DNA was amplified by PCR using specific primer sets, and the amplified PCR products were purified using FastGene gel/PCR extraction kit (NIPPON Genetics, Tokyo, Japan) according to the manufacturer's protocols. DNA methylation analyses were performed using the Quantification Tool for Methylation Analysis (QUMA) (http://quma.cdb.riken.jp/top/quma_main_j.html). The PCR primers used are listed in Table S7.

Statistical analyses

Significant differences between means for single comparisons were determined by Student's t test. Karyotype abnormalities were analyzed by chi-squared test. Significant difference was indicated by the asterisks, as follows: *$p < 0.05$; **$p < 0.01$; ***$p < 0.001$.

RESOURCE AVAILABILITY

Lead contact

Requests for further information and resources should be directed to and will be fulfilled by the lead contact, Takashi Shinohara (tshinoha@virus.kyoto-u.ac.jp).

Materials availability

This study did not generate new reagents.

Data and code availability

RNA-seq and RRBS data have been deposited in the Gene Expression Omnibus and accessible through accession numbers GSE280403, GSE280404, and GSE279277.

ACKNOWLEDGMENTS

This research utilized the Japanese Experiment Module of the International Space Station with the support of the JAXA. Financial support for this research was provided by the JAXA, AMED (JP24zf0127011), and MEXT (25H01357, 24H02045, 24K21291, 23K27727, 23H00399, and 23K20043). We thank Dr. Akira Higashibara, Mr. Tetsuya Sakashita, Mr. Yasuhiro Nakamura, Dr. Kazumi Koga, Mr. Yuho Amagai, Dr. Toru Shimadzu, Ms. Hiromi Sano, Ms. Ikuko Osada, Ms. Suzuki Tomoko, Mr. Daisuke Kawamura, Mr. Keisuke Aida, Mr. Hosogai Aki, and JAXA Flight Control Team and Payload Flight Control Team for help with performing the current study.

DECLARATION OF INTERESTS

The authors declare no competing interests.

SUPPLEMENTAL INFORMATION

Supplemental information can be found online at https://doi.org/10.1016/j.stemcr.2025.102602.

Received: May 2, 2025
Revised: July 20, 2025
Accepted: July 20, 2025
Published: August 14, 2025

REFERENCES

Ahrari, K., Omolaoye, T.S., Goswami, N., Alsuwaidi, H., and du Plessis, S.S. (2022). Effects of space flight on sperm function and integrity: a systematic review. Front. Physiol. *13*, 904375.

Aladjem, M.I., Spike, B.T., Rodewald, L.W., Hope, T.J., Klemm, M., Jaenisch, R., and Wahl, G.M. (1998). ES cells do not activate p53-dependent stress responses and undergo p53-independent apoptosis in response to DNA damage. Curr. Biol. *8*, 145–155.

An, L., Li, Y., Fan, Y., He, N., Ran, F., Qu, H., Wang, Y., Zhao, X., Ye, C., Jiang, Y., et al. (2019). The trends in global gene expression in mouse embryonic stem cells during spaceflight. Front. Genet. *10*, 768.

Amann, R.P., Deaver, D.R., Zirkin, B.R., Grills, G.S., Sapp, W.J., Veeramachaneni, D.N., Clemens, J.W., Banerjee, S.D., Folmer, J., Gruppi, C.M., et al. (1992). Effects of microgravity or simulated

launch on testicular function in rats. J. Appl. Physiol. *73*, 174S–185S.

Aschwood-Smith, M.J., and Friedmann, G.B. (1979). Lethal and chromosomal effects of freezing, thawing, storage time and X-irradiation on mammalian cells preserved at -196° in dimethyl sulfoxide. Cryobiology *16*, 132–140.

Avarbock, M.R., Brinster, C.J., and Brinster, R.L. (1996). Reconstitution of spermatogenesis from frozen spermatogonial stem cells. Nat. Med. *2*, 693–696.

Brinster, R.L., and Zimmermann, J.W. (1994). Spermatogenesis following male germ-cell transplantation. Proc. Natl. Acad. Sci. USA *91*, 11298–11302.

Cancedda, R., Liu, Y., Ruggiu, A., Tavella, S., Biticchi, R., Santucci, D., Schwartz, S., Ciparelli, P., Falcetti, G., Tenconi, C., et al. (2012). The mice drawer system (MDS) experiment and the space endurance record-breaking mice. PLoS One *7*, e32243.

Cirelli, E., De Domenico, E., Botti, F., Massoud, R., Geremia, R., and Grimaldi, P. (2017). Effect of microgravity on aromatase expression in Sertoli cells. Sci. Rep. *7*, 3469.

Clifton, D.K., and Brenner, W.J. (1983). The effect of x-irradiation on spermatogenesis in man. A comparison with the mouse. J. Androl. *4*, 387–392.

Cugia, G., Centis, F., Del Zotto, G., Lucarini, A., Argazzi, E., Zini, G., Valentini, M., Bono, M., Picardi, F., Stramigioli, S., et al. (2011). High survival of frozen cells irradiated with gamma radiation. Radiat. Protect. Dosim. *143*, 237–240.

Cunningham, G.R., and Huckins, C. (1978). Serum FSH, LH, and testosterone in ^{60}Co γ-irradiated male rats. Radiat. Res. *76*, 331–338.

De Felice, F., Marchetti, C., Marampon, F., Cascialli, G., Muzii, L., and Tombolini, V. (2019). Radiation effects on male fertility. Andrology *7*, 2–7.

de Rooij, D.G., and Russell, L.D. (2000). All you wanted to know about spermatogonia but were afraid to ask. J. Androl. *21*, 776–798.

de Waal, E., Yamazaki, Y., Ingale, P., Bartolomei, M., Yanagimachi, R., and McCarrey, J.R. (2012). Primary epimutations introduced during intracytoplasmic sperm injection (ICSI) are corrected by germline-specific epigenetic reprogramming. Proc. Natl. Acad. Sci. USA *109*, 4163–4168.

Esteves, S.C., Roque, M., Bedoschi, G., Haahr, T., and Humaidan, P. (2018). Intracytoplasmic sperm injection for male infertility and consequences for offspring. Nat. Rev. Urol. *15*, 535–562.

Fedorova, N. (1967). Spermatogenesis of the dogs Ugolyok and Veterok after their flight on board the satellite Kosmos 110. Kosm. Biol. Med. *1*, 28–31.

Fernández-González, R., Moreira, P.N., Pérez-Crespo, M., Sánchez-Martíín, M., Ramirez, M.A., Pericuesta, E., Bilbao, A., Bermejo-Alvarez, P., de Dios Hourcade, J., de Fonseca, F.R., et al. (2008). Long-term effects of mouse intracytoplasmic sperm injection with DNA-fragmented sperm on health and behavior of adult offspring. Biol. Reprod. *78*, 761–772.

Fleischman, R.A. (1993). From white spots to stem cells: the role of the Kit receptor in mammalian development. Trends Genet. *9*, 285–290.

Hargens, A.R., Steskal, J., Johansson, C., and Tipton, C.M. (1984). Tissue fluid shift, forelimb loading, and tail tension in tail-suspended rats – NASA Technical Reports Server (NTRS). Physiologist *27*.

Hong, Y., and Stambrook, P.J. (2004). Restoration of an absent G1 arrest and protection from apoptosis in embryonic stem cells after ionizing radiation. Proc. Natl. Acad. Sci. USA *101*, 14443–14448.

Holt, W.V. (2000). Fundamental aspects of sperm cryobiology: the importance of species and individual differences. Theriogenology *53*, 47–58.

Ishii, K., Ishiai, M., Morimoto, H., Kanatsu-Shinohara, M., Niwa, O., Takata, M., and Shinohara, T. (2014). The Trp53-Trp53inp1-Tnfrsf10b pathway regulates the radiation response of mouse spermatogonial stem cells. Stem Cell Rep. *3*, 676–689.

Ishii, K., Kanatsu-Shinohara, M., and Shinohara, T. (2014). Cell-cycle-dependent colonization of mouse spermatogonial stem cell transplantation into seminiferous tubules. J. Reprod. Dev. *60*, 37–46.

Kanatsu-Shinohara, M., Ogonuki, N., Inoue, K., Miki, H., Ogura, A., Toyokuni, S., and Shinohara, T. (2003). Long-term proliferation in culture and germline transmission of mouse male germline stem cells. Biol. Reprod. *69*, 612–616.

Kanatsu-Shinohara, M., Ogonuki, N., Inoue, K., Ogura, A., Toyokuni, S., and Shinohara, T. (2003). Restoration of fertility in infertile mice by transplantation of cryopreserved male germline stem cells. Hum. Reprod. *18*, 2660–2667.

Kanatsu-Shinohara, M., Inoue, K., Lee, J., Yoshimoto, M., Ogonuki, N., Miki, H., Baba, S., Kato, T., Kazuki, Y., Toyokuni, S., et al. (2004). Generation of pluripotent stem cells from neonatal mouse testis. Cell *119*, 1001–1012.

Kanatsu-Shinohara, M., Ogonuki, N., Iwano, T., Lee, J., Kazuki, Y., Inoue, K., Miki, H., Takehashi, M., Toyokuni, S., Shinkai, Y., et al. (2005). Genetic and epigenetic properties of mouse germline stem cells during long-term culture. Development *132*, 4155–4163.

Kanatsu-Shinohara, M., and Shinohara, T. (2013). Spermatogonial stem cell self-renewal and development. Annu. Rev. Cell Dev. Biol. *29*, 163–187.

Kanatsu-Shinohara, M., Naoki, H., and Shinohara, T. (2016). Nonrandom germline transmission of mouse spermatogonial stem cells. Dev. Cell *38*, 248–261.

Kanatsu-Shinohara, M., Ogonuki, N., Matoba, S., Ogura, A., and Shinohara, T. (2020). Autologous transplantation of spermatogonial stem cells restores fertility in congenitally infertile mice. Proc. Natl. Acad. Sci. USA *117*, 7837–7844.

Kanatsu-Shinohara, M., Shiromoto, Y., Ogonuki, N., Inoue, K., Hattori, S., Miura, K., Watanabe, N., Hasegawa, A., Mochida, K., Yamamoto, T., et al. (2023). Intracytoplasmic sperm injection induces transgenerational abnormalities in mice. J. Clin. Investig. *133*, e170140.

Kotovskaya, A.R., Koloteva, M.I., and Glebova, T.M. (2019). Tolerance of G-loads by a Russian cosmonaut and a NASA astronaut during the Soyuz space vehicle de-orbit after a 340-ay mission to the International Space Station. Hum. Physiol. *45*, 754–758.

Lei, X., Cao, Y., Ma, B., Zhang, Y., Ning, L., Qian, J., Zhang, L., Qu, Y., Zhang, T., Li, D., et al. (2020). Development of mouse preimplantation embryos in space. Natl. Sci. Rev. *7*, 1437–1446.

Lewon, M., Wang, Y., Peters, C., Peterson, M., Zheng, H., Wang, Z., Hayes, L., and Yan, W. (2020). Assessment of operant learning and memory in mice born through ICSI. Hum. Reprod. *35*, 2058–2071.

Mao, W., Huai, Y., An, L., Wang, X., Ru, K., Patil, S., Zhang, W., Ran, F., Chen, Z., and Qian, A. (2024). Microgravity inhibits cell proliferation and promotes senescence and apoptosis in embryonic stem cells. Space. Sci. Technol. *4*, 0104.

Masini, M.A., Albi, E., Barmo, C., Bonfiglio, T., Bruni, L., Canesi, L., Cataldi, S., Curcio, F., D'Amora, M., Ferri, I., et al. (2012). The impact of long-term exposure to space environment on adult mammalian organisms: a study on mouse thyroid and testis. PLoS One *7*, e35418.

Meistrich, M.L., and van Beek, M.E.A.B. (1993). Spermatogonial stem cells. In Cell and molecular biology of the testis, C.C. Desjardins and L.L. Ewing, eds. (New York: Oxford University Press), pp. 266–295.

Mishra, B., and Luderer, U. (2019). Reproductive hazards of space travel in women and men. Nat. Rev. Endocrinol. *15*, 713–730.

Miyazaki, T., Kanatsu-Shinohara, M., Ogonuki, N., Matoba, S., Ogura, A., Yabe-Nishimura, C., Zhang, H., Pommier, Y., Trumpp, A., and Shinohara, T. (2023). Glutamine protects mouse spermatogonial stem cells against NOX1-derived ROS for sustaining self-renewal division in vitro. Development *150*, dev201157.

Mori, Y., Ogonuki, N., Hasegawa, A., Kanatsu-Shinohara, M., Ogura, A., Wang, Y., McCarrey, J.R., and Shinohara, T. (2021). OGG1 protects mouse spermatogonial stem cells from reactive oxygen species in culture. Biol. Reprod. *104*, 706–716.

Nagamatsu, A., Murakami, K., Kitajo, K., Shimada, K., Kumagai, H., and Tawara, H. (2013). Area radiation monitoring on ISS increments 17 to 22 using PADLES in the Japanese experiment module Kibo. Radiat. Meas. *59*, 84–93.

Ogawa, T., Arechaga, J.M., Avarbock, M.R., and Brinster, R.L. (1997). Transplantation of testis germinal cells into mouse seminiferous tubules. Int. J. Dev. Biol. *41*, 111–122.

Plakhuta-Plakutina, G.I. (1977). State of spermatogenesis in rats flown aboard the biosatellite Cosmos-690. Aviat Space Environ. Med. *48*, 12–15.

Portugalov, V.V., Savina, E.A., Kaplansky, A.S., Yakovleva, V.I., Plakhuta-Plakutina, G.I., Pankova, A.S., Katunyan, P.I., Shubich, M. G., and Buvailo, S.A. (1976). Effect of space flight factors on the mammal: experimental-morphological study. Aviat Space Environ. Med. *47*, 813–816.

Sapp, W.J., Philpott, D.E., Williams, C.S., Kato, K., Stevenson, J., Vasquez, M., and Serova, L.V. (1990). Effects of spaceflight on the spermatogonial population of rat seminiferous epithelium. FASEB J. *4*, 101–104.

Shaka, S., Carpo, N., Tran, V., Cepeda, C., and Espinosa-Jeffrey, A. (2022). Microgravity alters neural stem cell division: implications for brain cancer research on earth and in space. Int. J. Mol. Sci. *23*, 14320.

Shinohara, T., Orwig, K.E., Avarbock, M.R., and Brinster, R.L. (2001). Remodeling of the postnatal testis is accompanied by dramatic changes in stem cell number and niche accessibility. Proc. Natl. Acad. Sci. USA *98*, 6186–6191.

Tou, J., Ronca, A., Grindeland, R., and Wade, C. (2002). Models to study gravitational biology of mammalian reproduction. Biol. Reprod. *67*, 1681–1687.

Wakayama, S., Kamada, Y., Yamanaka, K., Kohda, T., Suzuki, H., Shimazu, T., Tada, M.N., Osada, I., Nagamatsu, A., Kamimura, S., et al. (2017). Healthy offspring from freeze-dried mouse spermatozoa held on the international space station for 9 months. Proc. Natl. Acad. Sci. USA *114*, 5988–5993.

Wakayama, S., Ito, D., Kamada, Y., Shimazu, T., Suzuki, T., Nagamatsu, A., Araki, R., Ishikawa, T., Kamimura, S., Hirose, N., et al. (2021). Evaluating the long-term effect of space radiation on the reproductive normality of mammalian sperm preserved on the international space station. Sci. Adv. *7*, eabg5554.

Wakayama, S., Kikuchi, Y., Soejima, M., Hayashi, E., Ushigome, N., Yamazaki, C., Suzuki, T., Shimazu, T., Yamamori, T., Osada, I., et al. (2023). Effect of microgravity on mammalian embryo development evaluated at the Internatinal Space Station. iScience *26*, 108177.

Yoshida, K., Fujita, S.-I., Isotani, A., Kudo, T., Takahashi, S., Ikawa, M., Shiba, D., Shirakawa, M., Muratani, M., and Ishii, S. (2021). Intergenerational effect of short-term spaceflight in mice. iScience *24*, 102773.

Yoshida, K., Hada, M., Hayashi, M., Kizu, A., Kitada, K., Eguchi-Kasai, K., Kokubo, T., Teramura, T., Suzuki, H.H., Watanabe, H., et al. (2024). Transcriptome analysis by RNA sequencing of mouse embryonic stem cells stocked on International Space Station for 1584 days in frozen state after culture on the ground. Int. J. Mol. Sci. *25*, 3283.

Zemjanis, R., Gondos, B., Adey, W.R., and Cockett, A.T. (1970). Testicular degeneration in *Macaca nemistrina* induced by immobilization. Fertil. Steril. *21*, 335–340.

Stem Cell Reports, Volume *20*

Supplemental Information

Germline transmission of cryopreserved mouse spermatogonial stem cells maintained on the International Space Station

Mito Kanatsu-Shinohara, Takuya Yamamoto, Yusuke Shiromoto, Hiroko Morimoto, Tianjiao Liu, Tohru Yamamori, Tomokazu Yamasaki, and Takashi Shinohara

Figure S1

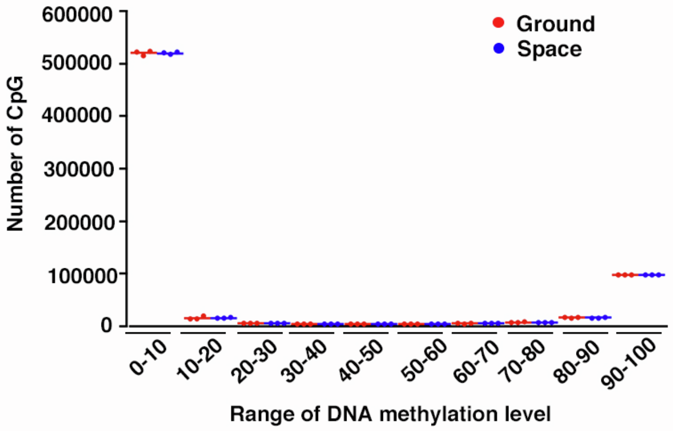

Figure S1. A histogram showing the number of CpG sites at any given DNA methylation level. The number of CpG sites was evaluated using 10% intervals for the rage of 0 to 100%. All promoter methylation sites were included in the determination of the mean of triplicate samples with a minimum read coverage of 10.

Figure S2

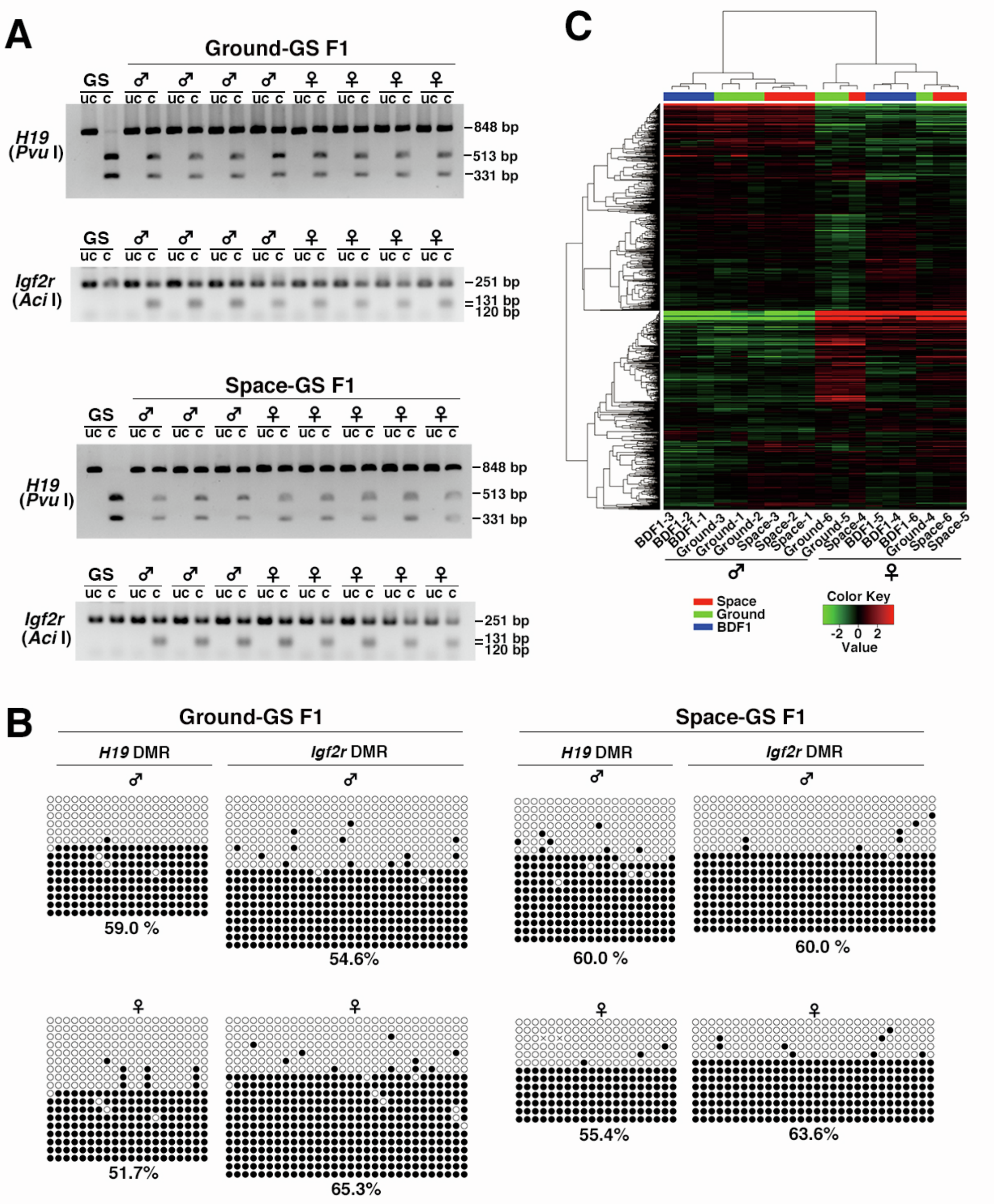

Figure S2. Analysis of genomic imprinting and gene expression in Space pups. (A) COBRA of tail DNA. (B) Bisulfite sequencing of tail DNA. (C) Heatmap of the top 1,000 genes with the most variable read counts in the RNAseq data of liver tissues.

Supplemental Methods

Culture conditions

GS cells used in the present study were previously described (Kanatsu-Shinohara et al., 2003). These cells were derived from C57BL6/Tg14(act-EGFP-OsbY01) mice that was bred into DBA/2 background (Okabe et al., 1997). The culture medium for GS cells included GDNF and FGF2, and cells were cultured on MEFs with 1% fetal bovine serum (Kanatsu-Shinohara et al., 2014). For cryopreservation, 1×10^6 GS cells were suspended in a cryopreservation solution (1ml; Cellbanker; DIA-IATRON, Tokyo, Japan). After pacing the cryotube (Nunc Biobanking and Cell Culture Cryogenic Tube) in a freezing container (CryoBoxes)(both from Thermo Fisher, MA), cells were frozen at -80°C. These tubes and containers were used in a previous study for ES cells (Yoshida et al., 2024). The samples and PADLES were launched to the ISS by SpaceX CRS-25 (SpX-25) on July 14, 2022, maintaining their frozen status and stored in MELFI at -95°C. After approximately 6 months, the samples were returned to the ground by Spade X CRS-26 (SpX-26) on January 11, 2023, maintaining their frozen status. At the ISS, the frozen vials were kept in MELFI. For thawing, the cryotubes were immersed in a water-bath at 37°C, and 10 ml of culture medium was added drop-wise. After washing by centrifugation, the cells were suspended in culture medium. For irradiation experiments, cells were frozen for 3 to 5 days.

RNA-seq

RNA-seq was carried out, as previously described (Kanatsu-Shinohara et al.,

2023). In brief, total RNA was purified using the RNeasy Plus Mini Kit (Qiagen,Valencia, CA) and cDNA libraries were generated using a TruSeq stranded mRNA library preparation kit (Illumina, San Diego, CA). Sequencing was performed using NextSeq550 (Illumina) with a single-read sequencing length of 76 bp (Kanatsu-Shinohara et al., 2023). The sequenced reads were mapped to the mm10 mouse reference genome using HISAT2, with the GENCODE M21 annotation gtf file after trimming adaptor sequences and low-quality bases with cutadapt-1.16 (Martin et al., 2011). Gene ontology analysis was performed using iDEP96 tool (http://bioinformatics.sdstate.edu/idep96/).

Immunostaining

Testis samples were fixed in 4% paraformaldehyde for 2 h at 4°C, embedded in Tissue-Tek OCT compound (Sakura Finetek, Tokyo, Japan) and used for cryosectioning. To block non-specific antibodies, sections were treated with 3% bovine serum albumin (BSA) and 10% donkey serum in phosphate-buffered saline (PBS) supplemented with 0.1% Tween 20 (PBST) for 1 h at room temperature. The sections were then incubated with indicated primary and secondary antibodies with 3 % BSA in PBST, overnight and for 1 h, respectively. Sections were washed with PBST. Rhodamine-labeled PNA (Vector Laboratories, Burlingame, CA) was used to detect the acrosome. Antibodies used are listed in Table S6. Hoechst 33342 (Sigma) was used for counterstaining.

Comet assay

The alkaline comet assay was performed, as described previously (Mori et al.,

2021). In brief, cells were incubated with hydrogen peroxide for 30 min on ice followed by the incubation in GS cell culture medium at 37°C for the indicated time. After centrifugation, cells were embedded in agarose on poly-L-lysine-coated glass slides (Matunami Glass, Osaka, Japan). The cells were lysed by incubating the slides at 4°C in lysis buffer [1% Trinton X-100, 0.5% N-Lauroyl sarcosine sodium salt, 2.5 M NaCl, 100 mM EDTA (pH 8.0), and 10 mM Tris–HCl (pH 10.0)]. After washing, the slides were immersed in pre-chilled electrophoresis buffer (0.3 M NaOH and 1 mM EDTA in water) for 40 min and electrophoresis was performed at 40 V for 50 min at 4°C. Then the samples were washed twice in 400 mM Tris–HCl (pH 7.0) at room temperature and soaked in ethanol for 5 min, followed by overnight drying at 37 ◦C. DNA was detected by ethidium bromide staining for 30 min at room temperature. After rinsing in PBS, slides were observed via confocal microscopy (Fluoview FV3000; Olympus, Tokyo, Japan), and quantified using by Fluoview FV31S-SW software.

Supplemental References

Kanatsu-Shinohara, M., Ogonuki, N., Inoue, K., Miki, H., Ogura, A., Toyokuni, S., and Shinohara, T. (2003). Long-term proliferation in culture and germline transmission of mouse male germline stem cells. Biol. Reprod. *69*, 612-616.

Kanatsu-Shinohara, M., Ogonuki, N., Matoba, S., Morimoto, H., Ogura, A., and Shinohara, T. (2014). Improved serum- and feeder-free culture of mouse germline stem cells. Biol. Reprod. *91*, 88.

Kanatsu-Shinohara M, Shiromoto Y, Ogonuki N, Inoue K, Hattori S, Miura K, Watanabe N, Hasegawa A, Mochida K, Yamamoto T, Miyakawa T, Ogura A, et al. (2023). Intracytoplasmic sperm injection induces transgenerational abnormalities in mice. J. Clin Invest. *133*, e170140.

Martin, M. (2011). Cutadapt removes adapter sequences from high-throughput sequencing reads. EMBnet.J. *17* (10).

Mori, Y., Ogonuki, N., Hasegawa, A., Kanatsu-Shinohara, M., Ogura, A., Wang, Y., McCarrey, J. R., and Shinohara, T. (2021). OGG1 protects mouse spermatogonial stem cells from reactive oxygen species in culture. Biol. Reprod. *104*, 706-716.

Okabe, M., Ikawa, M., Kominami, K., Nakanishi, T., and Nishimune, Y. (1997). 'Green mice' as a source of ubiquitous green cells. FEBS Lett. *407*, 313-319.

Yoshida, K., Hada, M., Hayashi, M., Kizu, A., Kitada, K., Eguchi-Kasai, K., Kokubo, T., Teramura, T., Suzuki, H. H., Watanabe, H., et al. (2024). Transcriptome analysis by RNA sequencing of mouse embryonic stem cells stocked on International Space Station for 1584 days in frozen state after culture on the ground. Int. J. Mol. Sci. 25, 3283.
